# Evaluation of local and systemic immune responses in pigs experimentally challenged with porcine reproductive and respiratory syndrome virus

**DOI:** 10.1186/s13567-020-00789-7

**Published:** 2020-05-13

**Authors:** Salik Nazki, Amina Khatun, Chang-Gi Jeong, Sameer ul Salam Mattoo, Suna Gu, Sim-In Lee, Seung-Chai Kim, Ji-Hyo Park, Myoun-Sik Yang, Bumseok Kim, Choi-Kyu Park, Sang-Myeong Lee, Won-Il Kim

**Affiliations:** 1 College of Veterinary Medicine, Jeonbuk National University, Iksan, South Korea; 2 Department of Pathology, Faculty of Animal Science and Veterinary Medicine, Sher-e-Bangla Agricultural University, Dhaka, 1207 Bangladesh; 3 Division of Biotechnology, Advanced Institute of Environment and Bioscience, College of Environmental & Biosource Science, Jeonbuk National University, Iksan, South Korea; 4 College of Veterinary Medicine, Kyungpook National University, Daegu, South Korea

## Abstract

The host-associated defence system responsible for the clearance of porcine reproductive and respiratory syndrome virus (PRRSV) from infected pigs is currently poorly understood. To better understand the dynamics of host–pathogen interactions, seventy-five of 100 pigs infected with PRRSV-JA142 and 25 control pigs were euthanized at 3, 10, 21, 28 and 35 days post-challenge (dpc). Blood, lung, bronchoalveolar lavage (BAL) and bronchial lymph node (BLN) samples were collected to evaluate the cellular immune responses. The humoral responses were evaluated by measuring the levels of anti-PRRSV IgG and serum virus-neutralizing (SVN) antibodies. Consequently, the highest viral loads in the sera and lungs of the infected pigs were detected between 3 and 10 dpc, and these resulted in moderate to mild interstitial pneumonia, which resolved accompanied by the clearance of most of the virus by 28 dpc. At peak viremia, the frequencies of alveolar macrophages in infected pigs were significantly decreased, whereas the monocyte-derived DC/macrophage and conventional DC frequencies were increased, and these effects coincided with the early induction of local T-cell responses and the presence of proinflammatory cytokines/chemokines in the lungs, BAL, and BLN as early as 10 dpc. Conversely, the systemic T-cell responses measured in the peripheral blood mononuclear cells were delayed and significantly induced only after the peak viremic stage between 3 and 10 dpc. Taken together, our results suggest that activation of immune responses in the lung could be the key elements for restraining PRRSV through the early induction of T-cell responses at the sites of virus replication.

## Introduction

Porcine reproductive and respiratory syndrome virus (PRRSV), a single-stranded positive-sense RNA virus with an approximate 15.4-kb genome, belongs to the genus *Betaarterivirus* of the family *Arteriviridae* (ICTV 2018). In pigs, PRRSV causes porcine reproductive and respiratory syndrome (PRRS), which is characterized by reproductive failure in breeding sows and severe respiratory distress in young and growing pigs [1]. PRRS results in colossal economic losses in the swine industry worldwide, and these losses are still observed three decades after its emergence in the United States and Europe. After the exposure of pigs to PRRSV, the virus replicates in alveolar macrophages (AM) and further spreads rapidly throughout the body via a lymphohematic route. This viral spread results in acute infection characterized by viremia that lasts for approximately 1 month [2], and a few studies have reported a nonviremic persistent infection of secondary lymphoid tissues lasting for approximately 150 days or longer [3]. In general, the viremia peaks at approximately 7–10 days post-infection (dpi) and is almost cleared by 28 dpi depending on the viral strain and age of the pigs [4, 5]. Additionally, the immune response against PRRSV depends on the strain, but the virus usually has immunosuppressive properties [4, 5], which leads to the increased susceptibility of pigs to secondary microbial infections [6]. The interactions between PRRSV and host immune responses have been widely studied, but most studies investigated systemic immune responses using PBMC and/or serum [7]. Previous studies have shown that interstitial pneumonia constitutes the major lung lesions in PRRSV-infected pigs and that significantly decreased numbers of alveolar macrophages are found in bronchoalveolar lavage (BAL) and lung parenchyma samples from PRRSV-infected pigs [8]. However, to the best of our knowledge, the kinetics of local immune responses in the lungs or lymph nodes during the course of infection compared with those of peripheral immune responses have not been previously studied. This information would provide a more in-depth understanding of the sequential activation of both immune compartments and the correlation between local or peripheral immune responses and virus clearance in infected pigs. As a result, achieving a comprehensive understanding of the immune responses against PRRSV infection remains an important goal in PRRSV research.

During PRRSV infection, the pig immune system is capable of escalating an immune response to ultimately clear the virus from the body [9]. For clearance, proper stimulation of the pig innate immune system is required to direct the development of protective adaptive immunity against PRRSV. Interestingly, the preferential sites for PRRSV replication are alveolar macrophages present in the lungs, which form the major component of the respiratory DC/macrophage network. This network is predominantly involved in sensing foreign antigens, controlling inflammation, and initiating the adaptive immune response [10]. Different DC subsets with specific functional specializations exist in the respiratory DC/macrophage network in the lungs and are reportedly resistant to PRRSV infection [8, 11]. However, upon activation, these DC travel to lymphoid tissues to present antigen to T lymphocytes and thereby serve as the link between innate and adaptive immunity [10, 11]. T cells, in turn, play a critical role in the development of anti-PRRSV immunity due to their cytotoxic effector functions in clearing infected cells from the body and developing and regulating antigen-specific immune responses [12]. However, whether the peripheral virus-specific immune response is appropriately correlated with the local immune response during infection, which could result in the precise use of the peripheral response as a surrogate for scrutinizing the local immune response during viral clearance, remains unclear. Therefore, discerning the local and peripheral immune responses during PRRSV infection is important for understanding the basic mechanism of viral clearance from the host.

Cytokines secreted by immune cells act on their targets in an autocrine, paracrine, and/or endocrine manner to prompt local and/or systemic immune responses. In porcine respiratory diseases, proinflammatory cytokines play a key role in activating and synchronizing the adaptive immune responses to clear the virus from the body [13]. However, the tissue damage caused by excessive production of these cytokines is controlled by the secretion of anti-inflammatory cytokines, which results in the maintenance of homeostasis in the body [14]. Moreover, effective instigation of the local inflammatory response in the lungs accompanied by significant changes in the proinflammatory cytokine levels in serum has been observed in pigs with respiratory diseases [15, 16]. Nevertheless, whether the local or the systemic cytokine/chemokine response plays the primary role in the clearance of PRRSV from pigs during the acute phase of infection remains unclear.

In this context, the present study aimed to investigate the trend of host immune responses against PRRSV infection during disease progression and to elucidate the innate and adaptive immunological mediators modulated by the PRRSV-JA142 strain both systemically in peripheral blood and locally in the bronchoalveolar lavage, lung parenchyma and bronchial lymph nodes (BLN) of infected pigs.

## Materials and methods

### Cells and viruses

MARC-145 cells, an African green monkey kidney cell line that is highly permissive to PRRSV [17], were used for virus propagation and assays. These cells were maintained in RPMI-1640 medium (Gibco^®^ RPMI-1640, Life Technologies, Carlsbad, CA, USA) supplemented with 10% heat-inactivated foetal bovine serum (FBS, Life Technologies), 2 mM l-glutamine, and an antibiotic–antimycotic cocktail (Anti-Anti, Life Technologies) containing 100 IU/mL penicillin, 100 µg/mL streptomycin, and 0.25 µg/mL Fungizone^®^ [amphotericin B] in a humidified chamber with 5% CO_2_ at 37 °C. In this manuscript, this medium is designated RPMI growth medium. The North American PRRSV-2 strain JA142 (GenBank: AY424271.1) was used in the present study.

### Animal study

**Figure 1:**
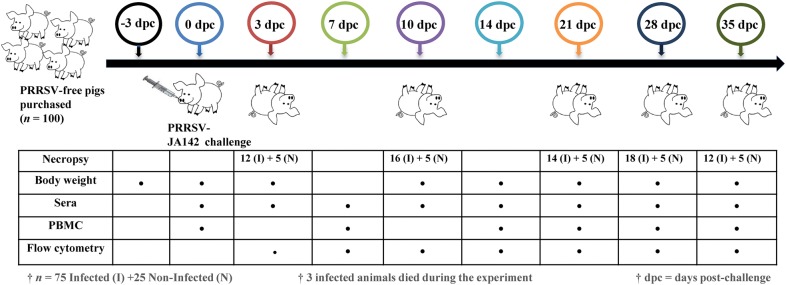
**Overview of the animal study.** PRRSV-negative piglets (*n* = 100) were purchased and acclimatized for 3 days. Seventy-five pigs were then infected, and 25 pigs were used as a negative control (NC). Pigs belonging to both the NC and infected (I) groups were euthanized at 3, 10, 21, 28 and 35 days post-challenge (dpc), and lung, bronchoalveolar lavage (BAL) and bronchial lymph node (BLN) samples were collected. Blood samples for sera and peripheral blood mononuclear cells (PBMC) were collected weekly post-challenge. The body weight of the pigs was also monitored from the day of purchase to the end of the experiment.

One hundred 4-week-old piglets purchased from a PRRSV-seronegative farm were randomly assigned to two groups and housed in separate animal rooms. After 3 days of acclimatization, the pigs in the infected group (*n* = 75) were intramuscularly inoculated with 2 mL of the PRRSV-JA142 strain (1 × 10^3^ TCID_50_/mL) diluted in sterile PBS. The control pigs (*n* = 25) remained uninfected. Feed and water were provided ad libitum to all the pigs. Five pigs from the control group and 12, 16, 14, 18 and 12 pigs from the infected group were humanely euthanized on days 3, 10, 21, 28 and 35 post-challenge (dpc), respectively. Euthanasia was performed by electrocution after the intramuscular injection of 2 mL of azaperone (40 mg/mL, StressGuard^®^, Dong Bang Inc., Seoul, South Korea). Three infected pigs died during the course of the experiment due to high fever and reduced body growth. An overview of the animal study is presented in Figure 1. After euthanasia, the lungs, trachea and bronchi were aseptically extracted and lavaged with 75 mL of sterile PBS. The collected lavage fluid was centrifuged at 1000 × *g* and room temperature for 10 min to separate the bronchoalveolar lavage fluid (BALF) and cells (BAL), and these samples, along with the lung parenchyma and BLN, were also used for immune cell analysis. These tissues and BALF were collected in tubes, snap-frozen using liquid nitrogen and stored immediately at −80 °C for RNA extraction and cytokine analysis, respectively. For histopathology, the lung tissues were also collected in 10% neutral-buffered formalin.

Blood was collected from the euthanized pigs at 3, 10, 21, 28 and 35 dpc, and the serum and PBMC were separated. In addition, blood samples from 42 pigs, including uninfected and infected pigs that were going to be euthanized on 28 and 35 dpc, were also collected at 0, 3, 7, 10, 14, 21 and 28 dpc, and the serum and PBMC were separated. The body weights of all the pigs were measured at 0 dpc, and the body weight gains of the euthanized pigs were measured at 3, 10, 21, 28 and 35 dpc.

### Quantification of the virus in serum and lung tissue

Serum viremia was measured at 0, 3, 7, 10, 14, 21, 28 and 35 dpc, and the viral load in the lungs was quantified in the euthanized pigs at 3, 10, 21, 28 and 35 dpc. Viral RNA was extracted from serum using a MagMAX viral RNA isolation kit (Ambion; Applied Biosystems, Life Technologies, Inc.) according to the manufacturer’s instructions. Real-time reverse transcription-polymerase chain reaction (RT-qPCR) employing the Prime-Q PCV2 PRRSV Detection Kit (Genet Bio, Daejeon, South Korea) was performed for the quantification of serum viremia. One-step RT-qPCR was performed in accordance with the manufacturer’s instructions. PCR amplification was performed using a model 7500 fast real-time PCR system (Applied Biosystems, Foster City, CA, USA). The cycling conditions were as follows: (i) cDNA synthesis for 20 min at 50 °C; (ii) 10-min predenaturation step at 95 °C; and (iii) 40 cycles of denaturation for 10 s at 95 °C and annealing/extension for 30 s at 60 °C. To calculate the amount of PRRSV in each sample, the Cq values were converted to virus titres (TCID_50_/mL) by generating a standard curve through the titration of PRRSV-2 strain JA142.

In addition, MARC-145 cells were used to quantify the virus titres in lung tissues using a microtitration infectivity assay [18]. Briefly, tissue homogenates (10% [weight/volume]) of finely chopped lung pieces were prepared in Dulbecco modified Eagle medium (DMEM) with antibiotics, and these mixtures were vortexed vigorously for 10–15 min and then centrifuged at ~4000 × *g* and 4 °C for 1 h. The collected supernatant was filtered through a 0.20-μm sterile syringe filter and used as an inoculum for the measurement of virus titres. The virus titres were calculated at 5 to 6 days post-inoculation based on the cytopathic effect (CPE) and are expressed as TCID_50_/mL [19].

### Pathological evaluation of the lungs

The lungs of the necropsied pigs in both groups were subjected to pathological evaluation on each day of necropsy. The microscopic lung lesions were given a score on a scale from 0 to 3 to reflect no lesion, mild interstitial pneumonia, moderate multifocal interstitial pneumonia, and severe interstitial pneumonia, respectively. The microscopic lesions were examined from five different lobes of the lungs, and the average value was ultimately utilized for scoring purposes.

### Anti-PRRSV-specific antibody detection

The serum samples from uninfected and challenged pigs were tested for anti-PRRSV antibody (IgG) using a commercially available ELISA kit (PRRS Ab ELISA 4.0; BioNote Inc., Hwaseong-si, Republic of Korea) according to the manufacturer’s instructions. Samples with an S/P ratio (the ratio of the net optical density of the test samples to the net optical density of the positive controls) ≥ 0.4 were considered to be positive for the PRRSV antibody.

The serum virus-neutralizing (SVN) antibodies were measured through a fluorescent focus neutralization (FFN)-based SVN assay with MARC-145 cells as described previously [20], with some modifications. After heat inactivation at 56 °C for 1 h, the serum samples were serially (twofold) diluted using RPMI-1640 growth medium. Two-hundred-microlitre mixtures were prepared by mixing each diluted serum sample with 10^3^ fluorescent focus-forming units per mL (FFU/mL) of PRRSV-JA142 at a ratio of 1:1 and were then incubated for 1 h at 37 °C in a humidified atmosphere with 5% CO_2_. Each mixture was transferred onto a monolayer of MARC-145 cells in 96-well plates and incubated for another 1 h at 37 °C. The medium was replaced with 200 µL of fresh RPMI growth medium per well and further incubated for 20 h at 37 °C. The cells were later fixed using ice-cold 80% (v/v) acetone, air-dried, and stained with mouse anti-PRRS NC Mab 4A5 (Median Diagnostic, Gangwondo, Korea) and FITC-conjugated goat anti-mouse IgG (h + l) (Bethyl Laboratories, TX, USA). Subsequently, the plates were washed at least three times with PBS and observed under a fluorescence microscope to examine the PRRSV-specific FFU. The SVN titre is expressed as the reciprocal of the highest dilution at which a 90% or higher reduction in the number of FFU was observed.

### Isolation of PBMC, BAL cells, and mononuclear cells from lymph nodes and lung tissues

PBMC were isolated from the blood samples (6 mL) by the density gradient method using Leucosep™ Centrifuge Tubes (Greiner Bio-One North America Inc., NC, USA) and Leucoprep™ Lymphocyte Separation Media (Greiner Bio-One North America Inc.) according to the manufacturers’ instructions. The blood samples were briefly stratified on Leucoprep™ solution at a ratio of 2:1 (blood:Leucoprep) and centrifuged at 1000 × *g* for 10 min. The purified PBMC were collected, washed twice with sterile PBS (pH 7.0) and resuspended in 0.5 mL of sterile PBS supplemented with 1% heat-inactivated FBS (Gibco, Carlsbad, CA, USA). Contaminating red blood cells (RBC) were removed by treatment with RBC lysis buffer (eBioscience, CA, USA).

For pathological evaluation, the right-sided lobes were clamped to collect specimens for RNA extraction and histopathology, and the left lobes of the lungs were used for BAL collection according to a previous study [21]. The lungs were lavaged with 50–75 mL of PBS containing 100 µg/mL ampicillin (USB Corporation Cleveland, OH, USA) and an antibiotic–antimycotic cocktail (Anti-Anti, Life Technologies), and the harvested fluid was centrifuged for 10 min at 1000 × *g*. The resulting supernatant was collected as BAL fluid (BALF), whereas the cell pellet (BAL cells) was washed three times with PBS after RBC lysis. The cells were resuspended in FACS buffer (3% FBS in phosphate-buffered saline and 0.02% sodium azide).

The BLN were passed through a 40-μm cell strainer (SPL Life Sciences, Pocheon, Korea) in PBS and then washed with FACS buffer according to a previous study [22]. The single cell suspension obtained was used for flow cytometric analysis.

Mononuclear cells from lung parenchyma were prepared based on a previous study [23], with few modifications. Briefly, lung tissue was collected, washed in sterile ice-cold PBS and suspended in serum-free RPMI media containing DNase I (25 U/mL, Sigma, St. Louis, MO, USA) and collagenase D (2 mg/mL, Roche Diagnostics, Mannheim, Germany). Single-cell suspensions were prepared using the gentleMACS Octo Dissociator (Miltenyi Biotec, San Diego, CA, USA) and incubated at 37 °C for 30 min. Subsequently, the cells were passed through a 40-μm cell strainer, washed, and resuspended in FACS buffer for flow cytometry analysis after RBC lysis.

Finally, the cells were counted with a Countess™ Automated Cell Counter (Invitrogen, Carlsbad, CA, USA), and their viability was tested by trypan blue (Sigma-Aldrich, St. Louis, MO, USA) exclusion [24].

### Flow cytometry

For cell surface staining, single-cell suspensions were incubated on ice for 30 min with specific antibodies as listed in Additional file 1, and the cells were then washed three times with FACS buffer. When necessary, secondary antibodies conjugated with fluorochrome were used. Natural killer (NK) cells, DC and macrophages required only cell surface staining, whereas the different subsets of T cells required intranuclear and intracellular staining.

Two subsets of NK cells have been phenotypically defined based on NKp46 marker expression: NKp46^+^ and NKp46^−^ NK cells [25, 26]. Following PBMC staining, a similar gating hierarchy was followed by excluding the unstained cells, doublets and CD3^+^ cells, and the CD3^−^ lymphocytes were further analysed for CD8α and NKp46 expression. Among CD3^−^ cells, two populations were found in the PBMC, namely, NKp46^+^ and NKp46^−^ NK cells, and both of these cells were CD8α^+^ (Additional file 2A).

BAL cells were subjected to cell surface staining for the CD163 surface marker, and the viability of these cells was analysed using propidium iodide (PI) staining (Additional file 2B). Additionally, DC and macrophages were segregated from the BAL cell population based on staining and gating strategies outlined previously (Additional file 2C) [10, 11]. From the MHC-II^+^ cell population, five phenotypically and functionally defined subpopulations were distinguished using the CD163 and CD172a (Sirpα) surface markers. Among the MHC-II^+^ cells, CD172a^+^/CD163^high^ cells were defined as AM, whereas CD172a^+^/CD163^int^, CD172a^+^/CD163^low^, CD172a^+^/CD163^−^ and CD172a^−^/CD163^−^ cells were defined as monocyte-derived macrophages (moMɸs), monocyte-derived dendritic cells (moDC), conventional dendritic cells 2 (cDC2) and conventional dendritic cells 1 (cDC1), respectively, based on a previous study [10].

Regulatory T cells (Tregs), which require intranuclear staining of FoxP3 after cell surface staining, were fixed with cold fixation/permeabilization buffer (eBioscience, Thermo Fisher Scientific, Seoul, Korea) at 4 °C for 30 min and were then stained for FoxP3 at 4 °C for 30 min. Based on a previously described staining and gating strategy [27], Tregs (CD25^+^FoxP3^+^ cells) were apparent among the CD4^+^CD8^−^ population (Additional file 2D).

The T-cell subsets subjected to intracellular staining were stained according to previous studies [28, 29], with few modifications. Briefly, single-cell suspensions were treated with a mixture of 1 × cell stimulation cocktail (eBioscience, Thermo Fisher Scientific, Seoul, Korea) and 1 × brefeldin A (eBioscience, Thermo Fisher Scientific, Seoul, Korea) in RPMI growth media and incubated at 37 °C in a humidified chamber with 5% CO_2_ for 4–5 h. The cells were then stained with antibodies for various cell surface markers in cold FACS buffer for 30 min at 4 °C, properly washed twice with cold FACS buffer, and fixed with intracellular (IC) fixation buffer (eBioscience, Thermo Fisher Scientific, Seoul, Korea) at 4 °C for 30 min. For intracellular staining, the cells were washed twice with permeabilization buffer (200 μL/well) and stained with cytokine-specific antibodies in cold permeabilization buffer at 4 °C for 30 min. Subsequently, the cells were washed twice with permeabilization buffer. The gating strategy employed for obtaining various T-cell phenotypes after the gating of singlet lymphocytes is demonstrated in Additional file 2E.

A 100-μL suspension of the stained cell populations in FACS buffer was run on an Accuri C6 flow cytometer (BD Accuri™ C6 Plus, BD Biosciences, MD, USA). BD Accuri™ C6 Plus software version 1.0.23.1 (BD Biosciences, MD, USA) was used to analyse the data after setting compensation settings according to monocolour and isotype control stains. The data are presented as percentages of all the cell subsets.

### Cytokine immunoassay

The cytokine levels in the sera and BALF of the uninfected and infected pigs at 3, 10 and 28 dpc were measured using a porcine-specific ProcartaPlex™ Multiplex Immunoassay (ThermoFisher Scientific, Vienna-1030, Austria) according to the manufacturer’s instructions. Magnetic microsphere technology based on porcine cytokine/chemokine antibody-immobilized magnetic beads was employed in the immunoassay for cytokine quantification [30]. The concentration of each cytokine was measured by running the samples on the Luminex^®^ 200™ system (Luminex Corporation, Austin, TX, USA). Appropriate standards provided in the kit were utilized to determine the concentration of each cytokine. The machine was verified and calibrated using a Luminex^®^ 100/200™ verification kit and a Luminex^®^ 100/200™ calibration kit (Luminex Corporation, Austin, TX, USA) prior to use.

### Data analysis

Graphical presentations of the data were prepared using GraphPad Prism 7.00 (GraphPad, San Diego, CA, USA), and the data were statistically analysed using SPSS Advanced Statistics 17.0 software (SPSS, Inc., Chicago, IL, USA). A nonparametric T-test (Mann–Whitney U test) was used to compare the viral loads in the lung tissues, the average daily weight gain (ADWG), the phenotypes of various cell subsets and the cytokine responses between two groups. The normalized dead CD163^+^ cells were analysed by repeated ANOVA (Tukey post hoc test) to determine the overall difference, and pairwise comparisons were also performed at different days post-challenge. Spearman rank correlation and linear regression were used to determine the associations between two parameters. Differences were considered statistically significant if *p *< 0.05 and are indicated by asterisks and different letters over the bars.

## Results

### PRRSV-JA142 infection leads to a reduced growth rate in pigs

**Figure 2:**
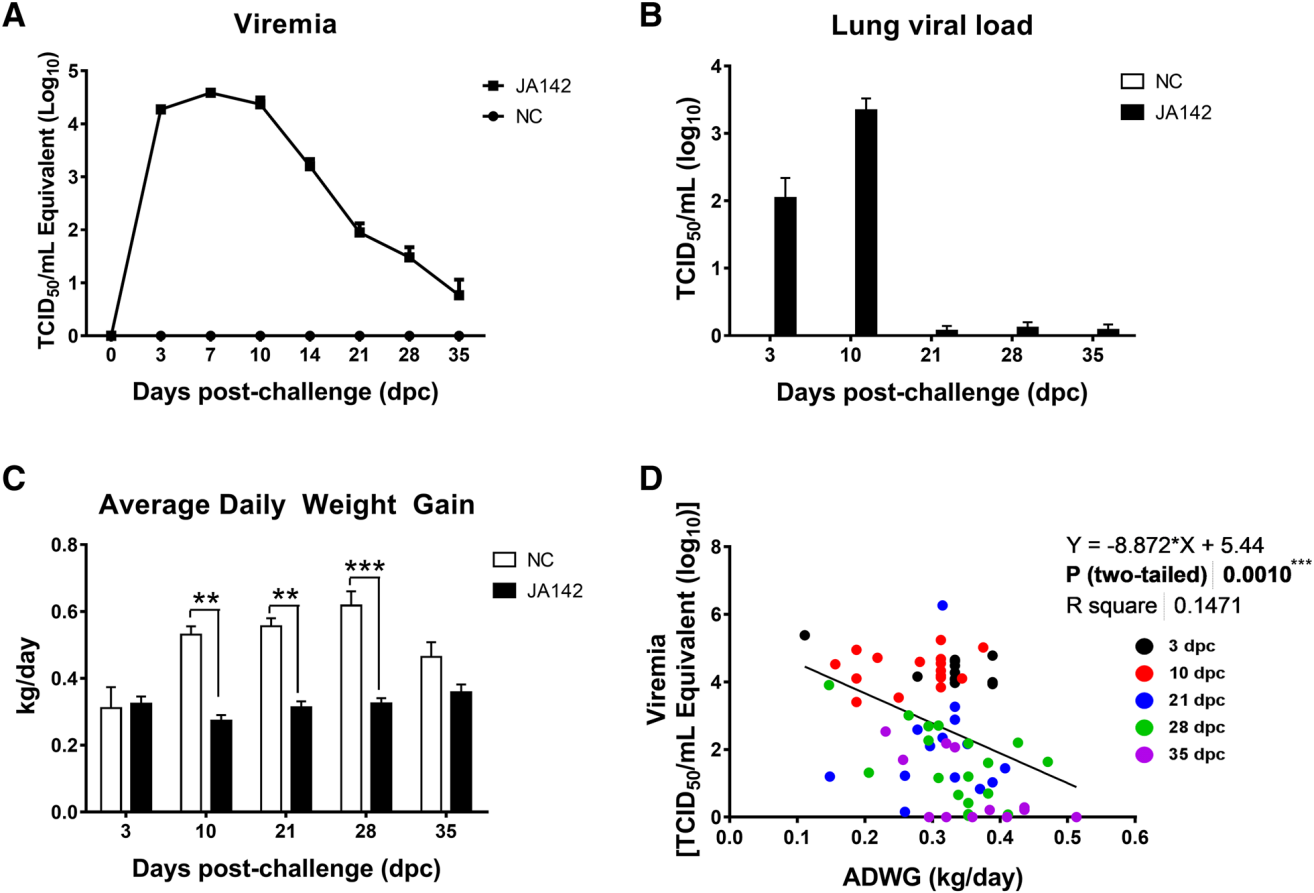
**Association of PRRSV-JA142 viral loads with the growth rate of pigs. A** The viral loads in sera obtained from negative control (NC) and infected (I) pigs at 0, 3, 7, 10, 14, 21, 28 and 28 dpc were quantified by real-time reverse transcription-PCR. The viral titres were calculated based on the standard curve of the threshold cycle number plotted against the known virus titre. **B** The residual viral lung loads at each necropsy day were quantified using MARC-145 cells based on a microtitration infectivity assay. **C** The body weight of the euthanized pigs was measured at −3, 3, 10, 21, 28 and 35 dpc, and the average daily weight gain (ADWG) was calculated. **D** The correlation between viremia and ADWG was tested during the course of infection. The bars represent the means, and the error bars represent the standard errors of the mean (SEM). Bars showing asterisks (*) represent values that differ significantly from each other (** indicates *p* ≤ 0.01 and *** indicates *p* ≤ 0.001).

The highest viremia and lung viral loads in PRRSV-JA142-challenged pigs were detected between 3 and 10 dpc (Figures 2A, B). The mean peak virus titre in serum at 7 dpc was recorded as 10^4.58^ TCID_50_/mL, whereas the mean peak live viral load in the lungs at 10 dpc, which was measured through the microtitration infectivity assay, was 10^3.34^ TCID_50_/mL. The virus was gradually cleared by 35 dpc, at which point, the serum and lung viral loads had decreased to mean values of 10^0.76^ and 10^0.08^ TCID_50_/mL, respectively. As expected, the control group maintained an uninfected state throughout the experiment. The effect of viremia on body weight gain was observed in the infected pigs by calculating the ADWG of the pigs (Figure 2C). The ADWG in the PRRSV-JA142-challenged pigs was significantly lower than that in the control pigs at 10, 21 and 28 dpc. The ADWG per control pig was recorded as 0.495 kg, whereas the ADWG of the infected pigs was reduced to 0.316 kg. As expected, the growth rate of the pigs was negatively affected by viremia following infection, and this negative correlation was significant (r = −0.3809; *p* ≤ 0.001) (Figure 2D).

### Moderate to severe interstitial pneumonia is observed in the infected pigs

**Figure 3:**
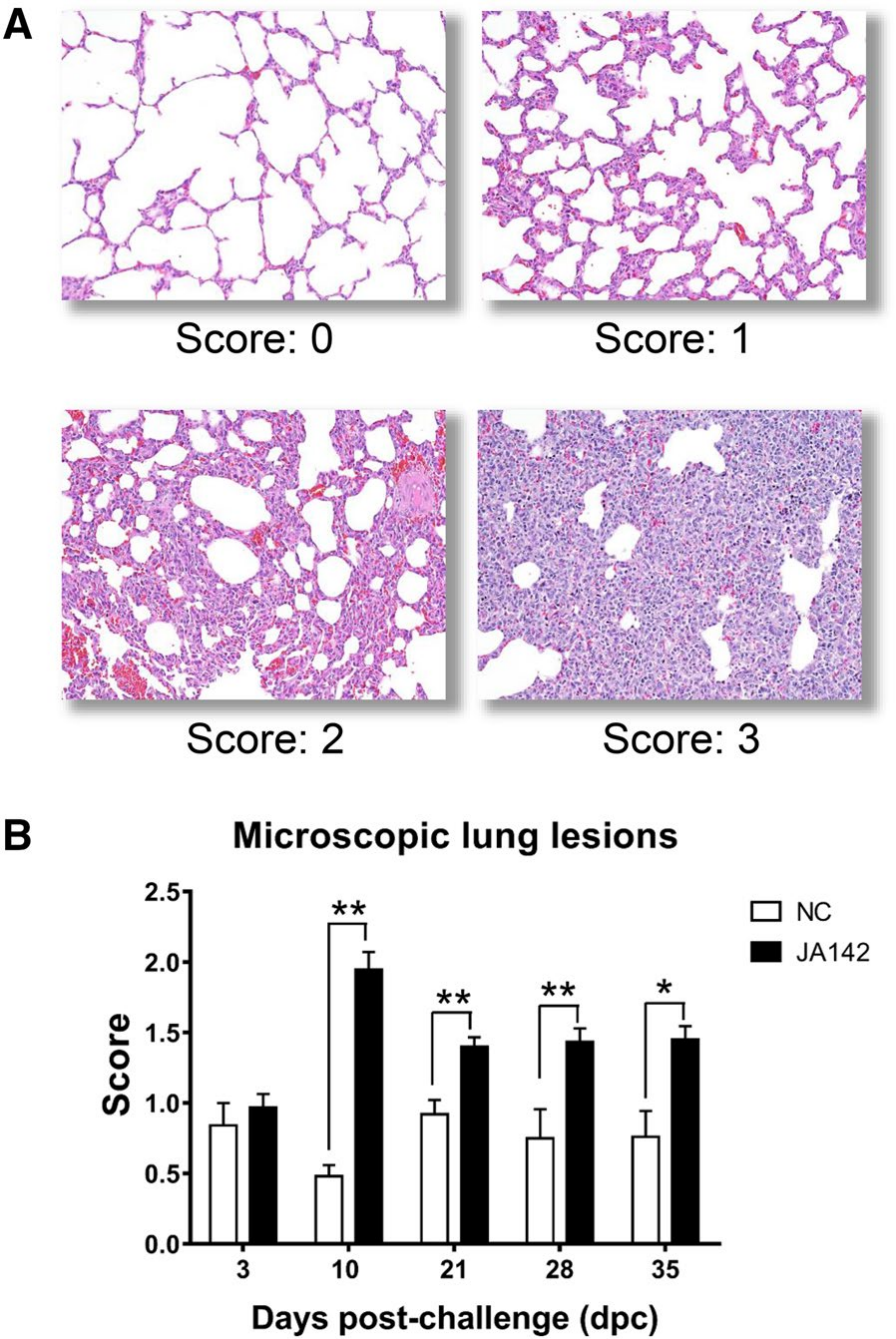
**Evaluation of microscopic lung pathology in the pigs on each necropsy day. A** Representative slides of H&E-stained lung sections used for scoring microscopic lesions in the uninfected (NC) and infected (I) pigs based on a 4-point scale from 0 to 3, with 0 representing no lesion, 1 representing mild interstitial pneumonia, 2 representing moderate/multifocal interstitial pneumonia and 3 representing severe interstitial pneumonia. **B** Mean microscopic lung lesion scores from pigs belonging to both groups on the particular day of necropsy. The error bars represent the SEM, and the bars showing asterisks represent values that differ significantly from each other (* indicates *p* ≤ 0.05 and ** indicates *p* ≤ 0.01).

Moderate to severe interstitial pneumonia with alveolar wall thickening due to type 2 pneumocyte proliferation and inflammatory cell infiltration was detected in the infected pigs during the period of peak viremia. Thus, the highest microscopic lung lesion score in the infected pigs was recorded at 10 dpc, and the scores of the lung lesions in these pigs decreased at later time points but remained at significantly higher levels compared with those in control pigs (Figures 3A, B).

### Kinetics of the anti-PRRSV antibody response

**Figure 4:**
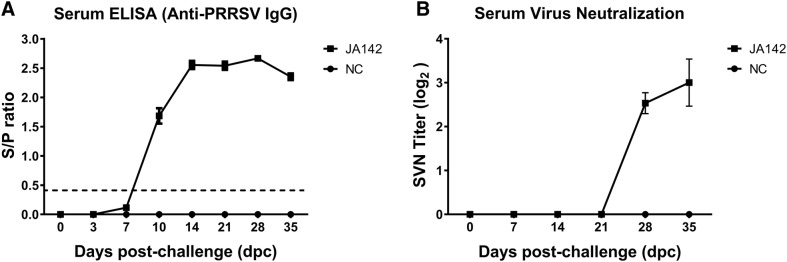
**Kinetics of the antibody response in pigs against PRRSV-JA142 infection. A** The PRRSV-specific antibody titre in pigs was measured by ELISA. The dashed line indicates the designated threshold value (S/P ratio of 0.4). **B** Weekly serum virus-neutralizing (SVN) antibody titres in uninfected (NC) and infected pigs obtained using homologous virus as the recall antigen. The bars represent the means, and the error bars represent the standard errors of the mean (SEM).

An ELISA based on the nucleocapsid (N) protein was employed to measure the PRRSV-specific antibody (IgG) response in the infected and noninfected pigs (Figure 4A). At 3 dpc, PRRSV-JA142-specific IgG were detected in the serum of the infected pigs. Additionally, the sample-to-positive (SP) value gradually increased in the infected group from 7 to 35 dpc, whereas in the control group, the pigs did not produce any PRRSV-specific IgG at any time point. A low SVN titre was observed in the challenged pigs at 28 and 35 dpc, when most of the virus was already cleared from the body (Figure 4B). In general, the SVN antibody responses predominantly appear at the later stages of infection, which is the phase at which most of the virus is cleared from the body, and might play a minor role in the clearance of virus.

### Early induction of NKp46^+^ and NKp46^**−**^ natural killer cells in PBMC post-infection

**Figure 5:**
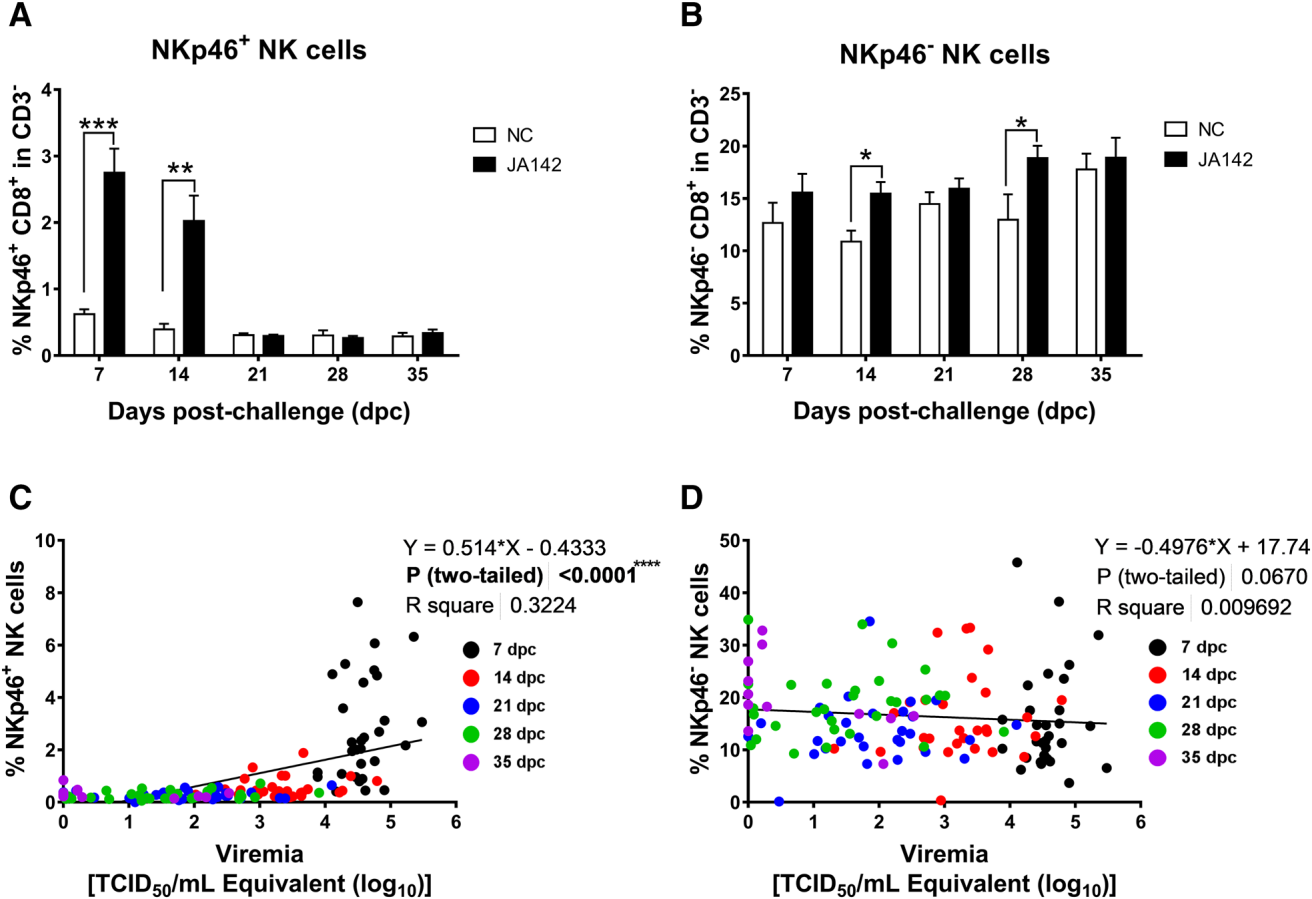
**Frequency of NK cells in the PBMC of negative control and infected pigs. A** The NKp46^+^ and **B** NKp46^−^ NK cell frequency in the PBMC of control (NC) and infected pigs over a period of 35 days post-challenge was tested, and the correlation between viremia and **C** NKp46^+^ and **D** NKp46^−^ NK cells was evaluated. An asterisk (*) over the bar indicates a significant difference (* indicates *p* ≤ 0.05, ** indicates *p* ≤ 0.01, *** indicates *p* ≤ 0.001 and **** indicates *p* ≤ 0.0001).

NK cells, which are a specialized subpopulation of lymphocytes, are the innate immune cells critically responsible for directly killing virus-infected cells, which ultimately leads to viral clearance in the host [31]. To observe the effect of PRRSV infection on NK cells in pigs, the frequencies of two different NK cell subsets in the PBMC population were analysed. The percentages of NKp46^−^ NK cells (CD8^+^NKp46^−^ in CD3^−^) in the PBMC populations of the uninfected and infected pigs were higher than those of NKp46^+^ NK cells (CD8^+^NKp46^+^ in CD3^−^) (Additional file 3A). Moreover, compared with the control pigs, the infected pigs exhibited a significantly (*p* ≤ 0.05) increased frequency of NKp46^+^ NK cells at the early time point of 7 dpc, whereas the frequency of NKp46^−^ NK cells was slightly increased at 7 dpc and significantly increased at 14 dpc. The frequency of NKp46^+^ NK cells returned to normal values at 21 dpc, but the frequency of NKp46^−^ NK cells in the infected pigs remained at significantly higher values up to 28 dpc (Figures 5A, B). The associations between NK cells in PBMC and serum viremia were evaluated post-infection (Figures 5C, D). The NKp46^+^ NK cell population revealed a significant (r = 0.6621; *p* < 0.0001) positive correlation with viremia; however, no statistically significant correlation between the levels of NKp46^−^ NK cells and viremia was observed.

### Dynamics of the respiratory dendritic cell/macrophage network are altered after infection

**Figure 6:**
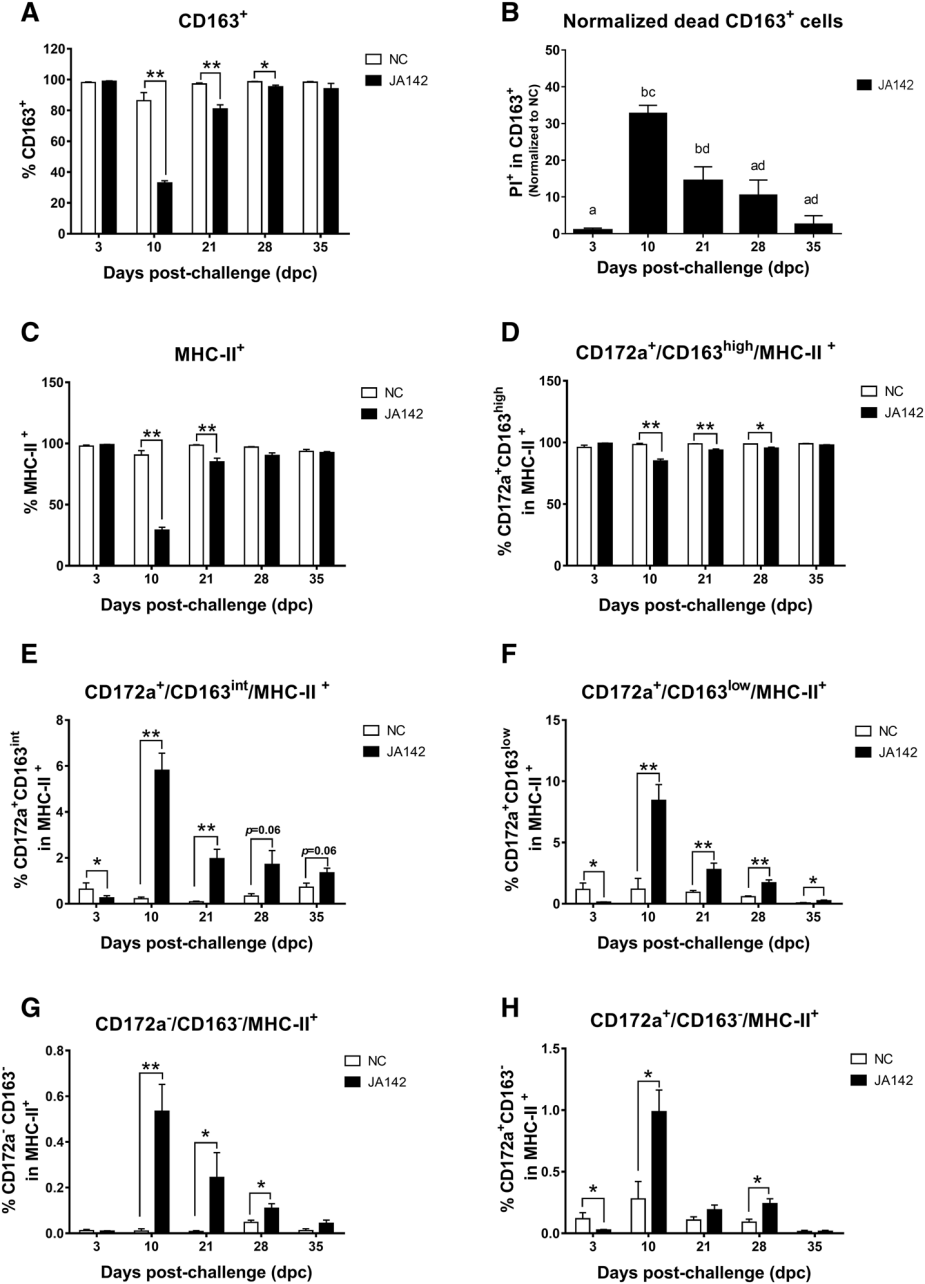
**Fluctuations in the frequencies of lung macrophage and dendritic cell phenotypes during infection.** Single-cell suspensions of BAL samples obtained from the right diaphragmatic and cardiac lobes of uninfected (NC) and infected pigs were stained for multicolour flow cytometry to detect fluctuations in the **A** total CD163^+^ cell population and the **B** normalized dead CD163^+^ cells at each necropsy day. The dynamics of the respiratory macrophage/DC network were observed by flow cytometric analysis of the BAL cells. **C** MHC-II^+^ cells were identified and further divided into five subsets based on the expression of CD163 and CD172a. Two subsets fitted to the macrophage lineage and were recognized as **D** CD172a^+^/CD163^high^/MHC-II^+^ (alveolar macrophages; AM) and **E** CD172a^+^/CD163^int^/MHC-II^+^ (monocyte-derived macrophages; moMɸ), whereas the remaining three subsets were classified as dendritic cells and were identified as **F** CD172a^+^/CD163^low^/MHC-II^+^ (monocyte-derived dendritic cells; moDCs), **G** CD172a^−^/CD163^−^/MHC-II^+^, and **H** CD172a^+^/CD163^−^/MHC-II^+^. The bars represent the means, and the error bars represent the standard errors of the mean (SEM). Bars showing asterisks and different letters represent values that differ significantly from each other (* indicates *p* ≤ 0.05, ** indicates *p* ≤ 0.01 and *** indicates *p* ≤ 0.001).

The receptors of host cells determine the cell tropism of PRRSV. Among others, CD163, a cysteine-rich scavenger receptor (SRCR), acts as the determinant receptor for PRRSV entry and infection [32, 33]. The BAL cells were subjected to CD163 staining, and the results show that although the virus did not affect the CD163^+^ cells until 3 dpc, the infected pigs exhibited a significant (*p* ≤ 0.05) reduction in the CD163^+^ cell percentage at 10 dpc, showing a gradual recovery with the decline in the viral loads at 35 dpc (Figure 6A). The reduction in the CD163^+^ cell population was attributed to the death of CD163^+^ cells, which was confirmed by observing the viability of these cells through PI staining. The mean frequencies of dead CD163^+^ cells in the infected pigs, which were normalized to those in the uninfected pigs, was significantly (*p* ≤ 0.05) higher at 10 and 21 dpc (32.7% and 14.35%, respectively) (Figure 6B).

**Figure 7:**
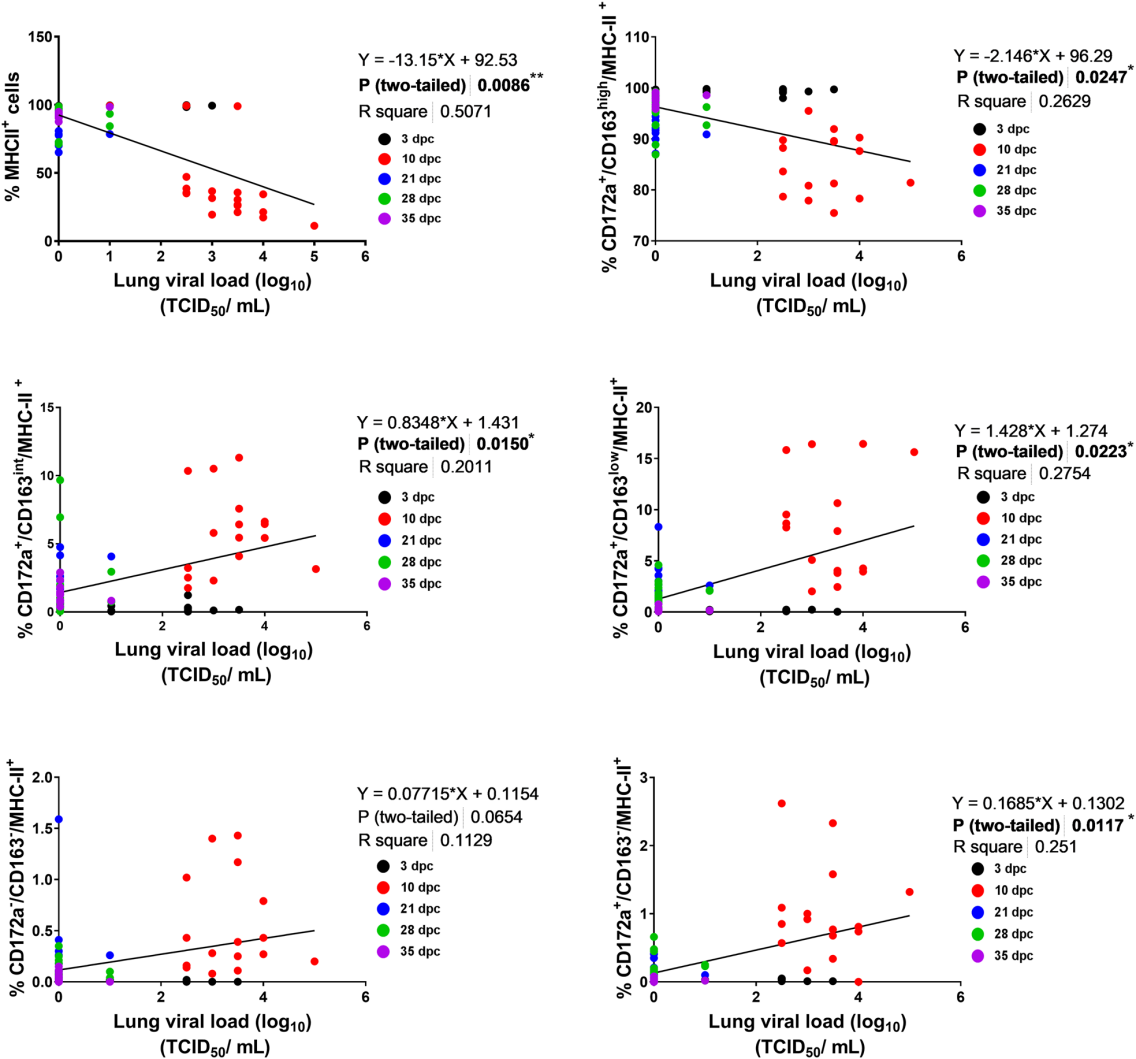
**Association between lung macrophage/dendritic cell phenotypes and lung viral loads in pigs after infection.** The correlation between different subsets of the DC/macrophage network in BAL cells and the lung viral loads was tested (* indicates *p* ≤ 0.05 and ** indicates *p* ≤ 0.01).

The class II major histocompatibility complex (MHC-II) is essential for the presentation of antigens to T cells and is constitutively expressed on macrophages and DC [34, 35]. The MHC-II^+^ cells were analysed to observe the changes in the DC/macrophage network in the lungs. The MHC-II^+^ cells, such as CD163^+^ cells, show significant (*p* ≤ 0.05) decreases at 10 and 21 dpc (Figure 6C). Interestingly, the percentage of AM (CD172a^+^/CD163^high^/MHC-II^+^ cells), which constituted the majority of MHC-II^+^ cells, decreased significantly at 10 dpc after infection (*p* ≤ 0.05) (Figure 6D). In contrast, the CD172a^+^/CD163^int^/MHC-II^+^ cell (moMɸ) and CD172a^+^/CD163^low^/MHC-II^+^ cell (moDC) frequencies were significantly (*p* ≤ 0.05) reduced in the infected pigs early during the infection process (3 dpc) (Figures 6E, F). However, the CD172a^+^/CD163^int^/MHC-II^+^ cell percentages in the infected pigs were significantly (*p* ≤ 0.05) increased at 10 and 21 dpc, and a higher (*p* ≤ 0.05) CD172a^+^/CD163^low^/MHC-II^+^ cell frequency was also detected at 10, 21, 28 and 35 dpc in the infected pigs compared with the control pigs. CD172a^+^/CD163^−^/MHC-II^+^ and CD172a^−^/CD163^−^/MHC-II^+^ cells were not frequently detected among BAL cells and accounted for less than 1% of the five subsets (Figures 6G, H). At 10 dpc, a significantly higher (*p* ≤ 0.05) percentage of these cell populations was observed in the infected pigs, and this value decreased with the reduction in the viral load and the recovery of AM. The associations between the lung viral loads and different subpopulations of macrophages and DC in BAL were evaluated after pooling the results of infected pigs at each time point (Figure 7). During the infection, MHC-II^+^ cells and AM displayed a significant negative correlation (*p* < 0.05) with lung viral loads; however, other subsets exhibited a significant (*p* < 0.05) positive correlation.

During infection, PRRSV-JA142 alters the dynamics of the respiratory DC/macrophage network by destroying AM while increasing the populations of antigen-presenting cells (APC), which bridge the gap between innate and adaptive immune systems by presenting the foreign antigen to T cells.

### Delayed peripheral T-cell responses in the infected pigs

**Figure 8:**
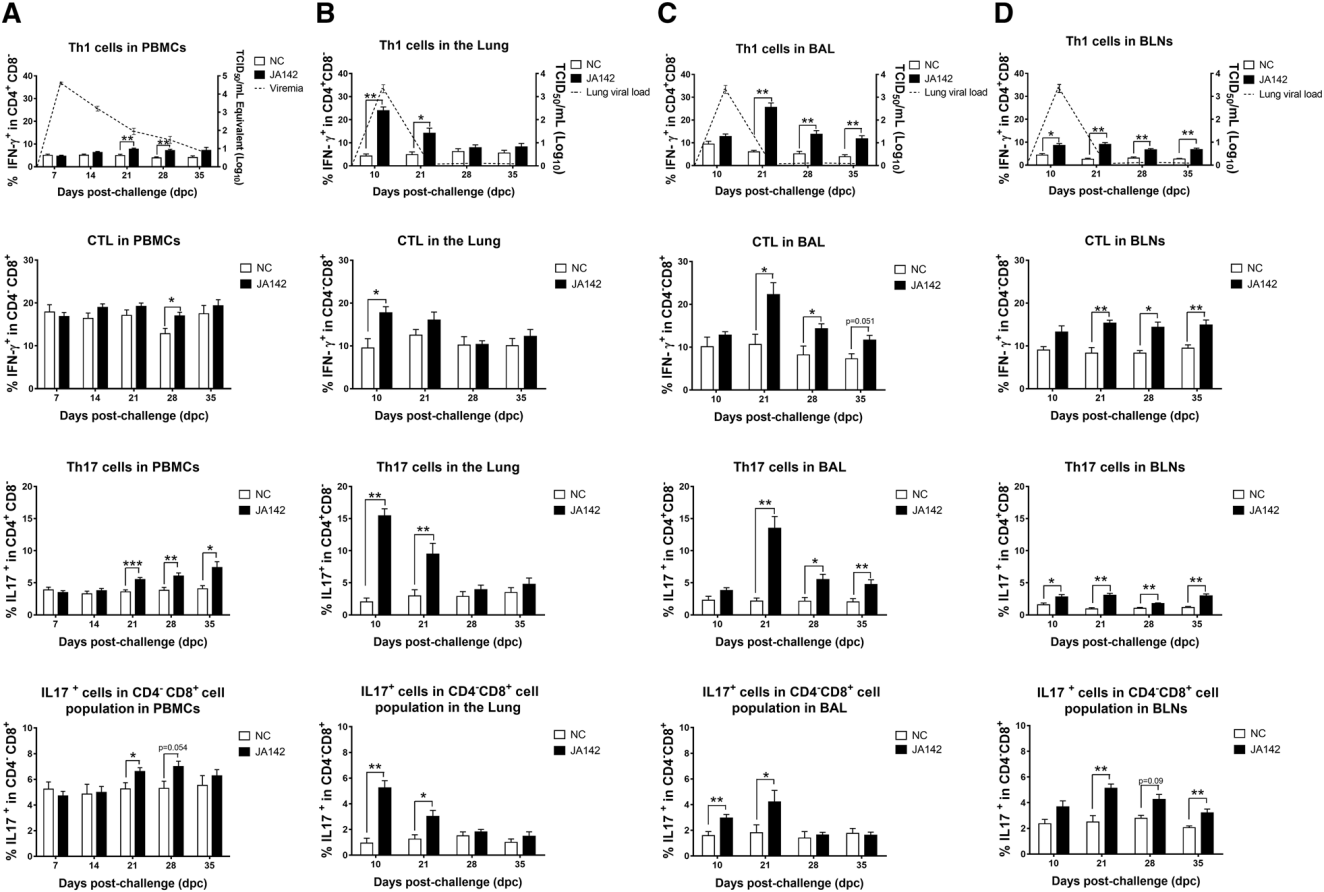
**Frequencies of T lymphocyte subsets in PBMC and tissues of uninfected and infected pigs.** Single-cell suspensions were immunostained to determine the frequency of various T-cell phenotypes in **A** PBMC, **B** lung parenchyma, **C** BAL and **D** BLN. Based on intracellular IFN-γ staining, the frequencies of Th1 cells (IFN-γ^+^ in CD4^+^CD8^−^) and CTL (IFN-γ^+^ in CD4^−^CD8^+^) were analysed. IL17 expression and its modulation was apparent in two types of cells: IL17-producing CD4^+^CD8^−^ cells and IL17-producing CD4^−^CD8^+^ cells. Each bar represents the average percentage of immune cells ± SEM. The asterisks (*) indicate statistically significant differences between the averages found for the uninfected (NC) pigs and those found for the challenged pigs at each time point (* indicates *p* ≤ 0.05, ** indicates *p* ≤ 0.01 and *** indicates *p* ≤ 0.001).

The peripheral immune response was measured by analysing the T-cell populations in the PBMC of uninfected and infected pigs at 7, 14, 21, 28 and 35 dpc (Additional file 3A). The Th1 (IFN-γ^+^ in CD4^+^CD8^−^) response in the infected pigs was significantly higher (*p* ≤ 0.05) than that in the control pigs at 21 dpc, and these responses continued to increase until 35 dpc. The cytotoxic T lymphocyte (CTL) (IFN-γ^+^ in CD4^−^CD8^+^) response in the infected pigs started to increase at 14 dpc and was significantly higher (*p* ≤ 0.05) compared with that in the control pigs at 28 dpc (Figure 8A). At 21 dpc, the Th17 (IL17^+^ in CD4^+^CD8^−^) cell response was significantly higher (*p* ≤ 0.05) in the infected pigs compared with the control pigs, and the response was further escalated at later time points. The IL-17-producing CD4^−^CD8^+^ cell population was significantly higher (*p* ≤ 0.05) in the infected pigs compared with uninfected pigs at 21 dpc. The delay in the induction of effector T cells was mainly perceived peripherally in blood.

### The local protective T-cell responses coincide with viral clearance

To observe the activation of the local adaptive immune responses by innate immune cells at the sites of replication and the persistence of PRRSV, the T-cell phenotypes in the BLN, BAL and lung parenchyma of the euthanized control and infected pigs were analysed at 10, 21, 28 and 35 dpc. The percentages of various immune cells in the lungs, BAL and BLN of uninfected and PRRSV-JA142-infected pigs at different stages of infection are summarized in Additional files 3B, C, and D. Overall, various T-cell responses were significantly induced in the lungs as early as 10 dpc, and significant responses in BAL and BLN were first detected at 10 dpc and were maintained until 35 dpc. The Th1 cell (IFN-γ^+^ in CD4^+^CD8^−^) frequency in all the local tissues was significantly (*p* ≤ 0.05) induced in the challenged pigs as early as 10 dpc, and the frequency in BAL cells tended to be higher. The CTL (IFN-γ ^+^ in CD4^−^CD8^+^) frequency was significantly (*p* ≤ 0.05) higher in the lungs of the infected pigs compared with that of the control pigs at 10 dpc, whereas the frequency in BLN and BAL cells was higher at the early time point and significantly higher at 21 dpc. The PRRSV-JA142-infected pigs also displayed a higher induction (*p* ≤ 0.05) of Th17 cells (IL17^+^ in CD4^+^CD8^−^) in the lymph nodes and lung tissues at 10 dpc, whereas a slight increase in these cells was observed in BAL cells. The IL-17-producing CD4^−^CD8^+^ population was significantly (*p* ≤ 0.05) induced in the lungs and BAL cells of the infected pigs at 10 dpc, whereas in BLN, this cell population showed a slight increase at 10 dpc and a significant increase (*p* ≤ 0.05) at 21 dpc (Figures 8B–D). Therefore, compared with the weak and delayed peripheral responses, early and effective cellular immune responses were triggered in local tissues by PRRSV-JA142 infection.

### PRRSV-JA142 does not induce regulatory T cells in infected pigs

**Figure 9:**
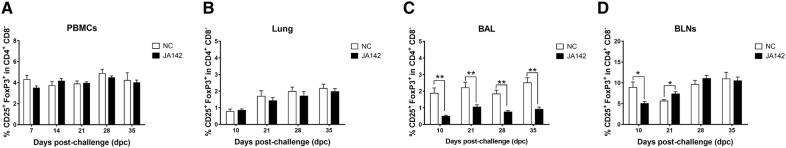
**Frequencies of regulatory T-cell populations.** The percentages of CD25^+^Foxp3^+^ cells in the CD4^+^CD8^−^ T lymphocyte populations in PBMC, BLN, BAL and lung parenchyma single-cell suspensions were enumerated by flow cytometry. The frequency of the Treg subpopulations in the negative control and infected pigs at each time point during the course of infection is presented in the graphs. The data represent the mean ± SEM for each population, and the asterisk (*) indicates a statistically significant difference between the averages found for the negative control (NC) pigs and those obtained for the challenged pigs at each time point (* indicates *p* ≤ 0.05 and ** indicates *p* ≤ 0.01).

Tregs are well known for their immunosuppressive activities, and previous studies have demonstrated that PRRSV infection induces Treg responses that might be responsible for ineffective adaptive immune responses [36–38]. Unlike previous studies, no upregulation of Tregs (CD25^+^Foxp3^+^ in CD4^+^CD8^−^) was detected in PBMC isolated from PRRSV-infected pigs. Moreover, Tregs were significantly (*p* ≤ 0.05) reduced in the BAL throughout the course of infection, but no such decline was observed in the lungs (Figure 9). However, the Treg frequencies in BLN of the infected pigs initially exhibited a significant decline at 10 dpc but then increased significantly at 21 dpc.

### The localized induction of proinflammatory cytokine responses was pragmatic at peak viremia

**Figure 10:**
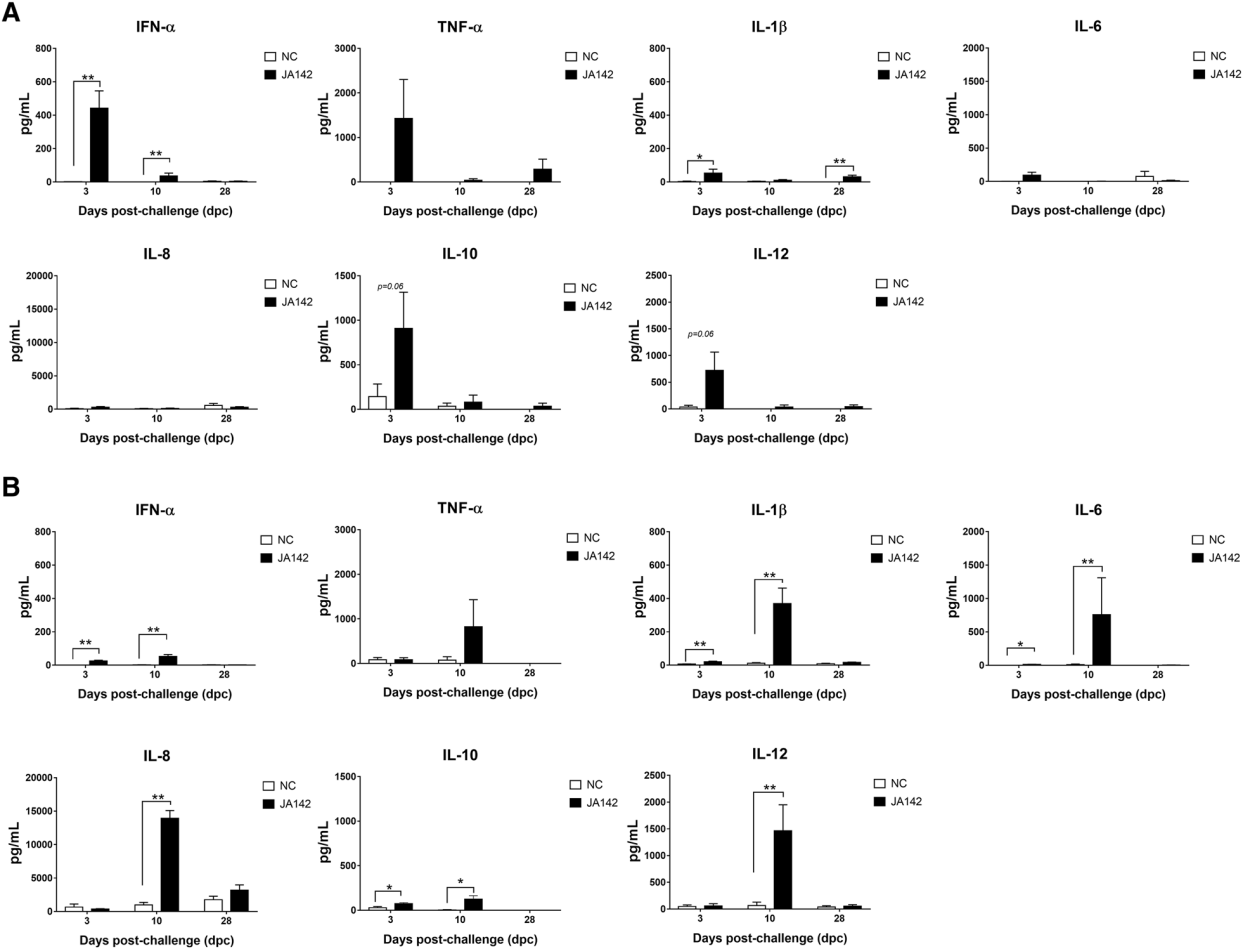
**Local and systemic levels of cytokines/chemokines in the pigs.** The concentrations of cytokines/chemokines in the **A** sera and **B** BALF of pigs belonging to both groups at 3, 10, and 28 dpc were measured using a multiplex Luminex-based cytokine immunoassay. The bars in the graphs represent the mean ± SEM of the cytokine levels, and the asterisks (*) indicate a statistically significant difference between the averages found for the uninfected (NC) pigs and those obtained for the infected pigs at each time point (* indicates *p* ≤ 0.05 and ** indicates *p* ≤ 0.01).

The levels of seven different innate and adaptive cytokine/chemokine proteins in the sera and BALF at 3, 10 and 28 dpc were compared between the uninfected and infected pigs (Figure 10). The results reveal that interferon-α (IFN-α) was significantly (*p* ≤ 0.05) induced in the sera and BALF of the infected pigs at 3 dpc. However, at peak viremia (10 dpc), the IFN-α level was reduced peripherally in the sera of the infected pigs, whereas the cytokine level was elevated locally in the BALF. Nevertheless, significant increases in the level of this cytokine (*p* ≤ 0.05) were maintained both locally and systemically in the infected pigs compared with the uninfected pigs. Furthermore, proinflammatory cytokines/chemokines, such as tumour necrosis factor-α (TNF-α), interleukin-1β (IL-1β), IL-6, IL-8 and IL-12, show a similar pattern of early induction at 3 dpc in the sera of the infected pigs followed by decreases as viremia increased. In the sera, significant (*p* ≤ 0.05) changes in IL-1β were only observed in the infected pigs at 3 and 28 dpc. However, significant (*p* ≤ 0.05) induction of IL-1β and IL-6 locally in the BALF was observed in the infected pigs at 3 dpc. In addition, elevations in all the proinflammatory cytokine/chemokine levels were observed in the infected pigs as the viral load increased, and significant increases in the levels of IL-1β, IL-6, IL-8 and IL-12 were observed in the infected pigs compared with the uninfected pigs at 10 dpc. Moreover, IL-10, an anti-inflammatory cytokine, was significantly induced (*p* ≤ 0.05) at 3 and 10 dpc in the BALF of the infected pigs. Overall, the clearance of the virus from the pigs also coincided with the increased secretion of anti-viral and proinflammatory cytokines locally in the BALF of PRRSV-infected pigs, although no considerable shifts were detected in the serum.

## Discussion

Despite exhaustive research, the understanding of the protective immune responses against PRRSV in pigs remains limited. Previous studies have mainly focused on certain aspects of immune responses and/or have used a narrow time window to study the immune responses during PRRSV infection; therefore, the available data offer a limited picture of the host defence system. To the best of our knowledge, the present study constitutes the first investigation of various aspects of immune responses, such as local vs. systemic and innate *vs* adaptive, during the course of PRRSV infection to obtain a broader picture of the host defence against PRRSV. Here, we demonstrate the critical role of local immune responses in the clearance of PRRSV from pigs due to the early induction of DC/macrophage subsets, the activation of protective T cells in local lymphoid and lung tissues, and the stimulation of proinflammatory cytokines locally in the lungs.

In pigs that were intramuscularly inoculated with the biologically characterized PRRSV-JA142 strain [20], the viral titre reached its peak value in the serum and lungs between 3 and 10 dpc. In addition, moderate to severe interstitial pneumonia and a significant decrease in the ADWG, which are characteristics of PRRSV infection [39], were detected in the challenged pigs by 10 dpc. Mild histopathological lesions (with a lesion score lower than 1) characterized by alveolar wall thickening due to type 2 pneumocyte proliferation were also observed in some of the uninfected healthy pigs that were free of other respiratory pathogens. These observations were likely obtained due to the stress induced by environmental factors, such as housing conditions, weaning, individual space, and ambient temperature variations, as previously reported [40, 41].

Similar to previous reports [7], the PRRSV-JA142 strain induced delayed (≥ 28 dpc) and weak (≤ 8) SVN titres in infected pigs. An SVN antibody titre of 32 offers sterilizing immunity, whereas a titre greater than 8 is considered protective against PRRSV infection [42]. The anti-PRRSV antibody response induced by PRRSV-JA142 in the infected pigs crossed the threshold of 0.4 (S/P ratio) between 3 and 10 dpc, and this response was in accordance with the response produced by other PRRSV strains [5], but the role of these antibodies in protection against PRRSV infection is unknown [12].

NK cells, an important component of the innate host defence system, play a critical role in the resolution of viral infections [31]. In general, the potential roles of NK cells in relation to PRRSV immunity are poorly understood [43, 44]. In the current study, two defined NK cell subpopulations were distinguished in PBMC based on an approach similar to one previously described [25]. Similar to previous observations, the NKp46^+^ NK cell frequency in PBMC collected from both control and infected pigs was lower than that of NKp46^−^ cells. NKp46^+^ and the NKp46^−^ NK cells execute analogous cytolytic activities but produce different levels of IFN-γ, with NKp46^+^ NK cells producing higher amounts of IFN-γ. Moreover, NKp46 can be expressed in NKp46^−^ NK cells after stimulation with interleukins (IL)-2, IL-12 and IL-18 [25]. In the current study, early increases in the frequencies of NKp46^+^ and NKp46^−^ NK cells in PRRSV-JA142-challenged pigs were clearly detected. Previous studies have shown similar increases in the CD3^−^CD4^−^CD8^+^ cell population after infection with different strains of PRRSV [7, 45]. Induced proliferation of NK cells has also been perceived after influenza infection, suggesting that this subset of lymphocytes is capable of antigen-specific clonal expansion [46]. PRRSV, however, significantly suppresses NK cell-mediated cytotoxicity to evade the host immune response [45], but these previous studies did not consider the subsets of NK cells. The specific action of different subsets of NK cells on PRRSV has not yet been explored, and further studies are needed to explain the role of NK cells in the containment of the virus during infection.

The main targets of PRRSV are cells belonging to the monocyte and macrophage lineages, particularly AM. However, DC are also reportedly vulnerable to PRRSV infection [47]. The DC/macrophage network, which senses the foreign antigen and initiates the immune response, constitutes one of the main components of the respiratory immune system [10]. It is plausible to expect that the viral infection of these cells alters this network and thus affects downstream immune responses. Therefore, dissecting how these immune cells respond to PRRSV infection is important to better understand the nature of PRRS. Until now, limited studies have investigated the alterations in the respiratory DC/macrophage network in pigs during disease progression [8, 10], and the association of the alteration in this immune network with the T-cell response has not been reported. Here, we attempted to explore the dynamics of this important host defence mechanism in relation to PRRSV infection. During infection, PRRSV replicates in the CD163^+^ cells in the lungs and induces apoptosis at the early stages of infection [33]. In the current study, a flow cytometric analysis of BAL cells revealed that PRRSV-JA142 decreased the CD163^+^ cell population in the infected pigs at the time when peak viral loads were detected. After the viral load decreased, the CD163^+^ cell population recovered to the normal levels. PI staining of the CD163^+^ BAL cells revealed that the decrease in the CD163^+^ cell population was due to cell death. A similar reduction in the macrophage population after PRRSV-1 infection was previously reported [48]. Following the strategy described previously [11], different subsets of DC and macrophages were identified in BAL cells, and the changes in the populations of these subsets during disease progression were studied. Previous reports verify that PRRSV infection impairs DC function directly by downregulating MHC-II expression [24, 49]. Intriguingly, among the five cell subsets in MHC-II^+^ cells, only the CD172a^+^/CD163^high^/MHC-II^+^ (AM) cell population decreased significantly at peak viremia, revealing a significant negative correlation between the population of these cells and the viral loads in the lungs, whereas significant positive correlations were found for the other subset populations. The increases in the CD172a^+^/CD163^int^/MHC-II^+^ and CD172a^+^/CD163^low^/MHC-II^+^ cell populations can be attributed to the higher influx of monocytes and their differentiation in the lungs during inflammation. Moreover, CD172a^+^/CD163^−^/MHC-II^+^ and CD172a^−^/CD163^−^/MHC-II^+^ cells exhibit strong migration and antigen-presenting capabilities [10], which can be credited to the increased influx of these cells into the lungs during the course of infection. Our results were in agreement with those obtained in previous studies, which found that the populations of these cells are increased in the BAL after PRRSV-1 infection [8]. Interestingly, cDC1s activate allogenic naïve T cells and aid induction of the Th1 response, whereas cDC2s induce a Th2 response in pigs [10]. After sensing antigen, the mature migrating DC recruit NK cells to drain LN, thus providing an early source of IFN-γ and thereby promoting Th1 polarization [50]. Thus, DC work together with NK cells to regulate innate immunity and further dictate the direction and intensity of the adaptive immune response [51].

The effective immune response to counter viral infections is governed by the proper activation of T lymphocytes by APC [52]. In the current study, we analysed the dynamics of T lymphocytes in PBMC, lung parenchyma, BAL, and BLN to detect the central cause of the clearance of PRRSV from the body. Delayed induction of Th1, Th17 and CTL responses was observed in the PBMC of the infected pigs after 21 dpc, when most of the virus had been cleared from the blood, whereas in BLN, the virus persists for a longer period, which leads to early and sustained (> 35 dpc) induction of T cell responses. Previous studies also found a delayed induction of effector T cells in the peripheral blood of pigs infected with PRRSV [7, 29]. Intriguingly, significant increases in the frequencies of these T lymphocyte subpopulations were detected in the lung parenchyma and lymphoid tissues of the infected pigs at early stages of infection (10 dpc), when the virus levels were at their peak in the body. In agreement with our findings, a higher frequency of T-helper cells and CTL has been observed in local tissues, such as the BAL, lymph nodes and lung parenchyma, of pigs after swine influenza infection, and this induction has been linked to viral clearance [23]. The local cell-mediated immune responses are considered crucial for the clearance of the influenza virus [53]. Moreover, during human respiratory syncytial virus infection, increased populations of CD4^+^ and CD8^+^ T cells exhibiting effector functions have been observed in the BAL of infected patients [54]. A recent study investigating T-cell proliferation in PRRSV-2-infected PBMC at 28 dpi and the expression of lymph node-homing receptors revealed that the T-helper cell response plays a main role in viral clearance and that the CTL response is strongest at the site of infection [55]. In addition, the induction of Th17 cells in local tissues has also been found to be essential for the resolution of respiratory infections [56]. In the present study, the cell responses in BLN and BAL were found to be significantly increased from 10 or 21 dpc and were maintained until 35 dpc, when the virus was completely cleared. Therefore, it can be concluded that local T-cell responses in the lungs, BLN and BAL are induced markedly faster than systemic responses and are maintained at significantly high levels, even after virus clearance, substantiating the critical role of local immune responses in the clearance of PRRSV from pigs.

The Treg lineage is responsible for the maintenance of homeostasis in the immune system by suppressing the activation of various immune cells, including other T cells, NK cells and DC [57]. Treg play a vital role in the pathogenesis of some viral infections that result in severe inflammatory lesions, such as influenza [23]. However, the Treg response in pigs after PRRSV infection is controversial. Although some studies have revealed increased frequencies of Tregs after PRRSV infection [36, 37, 58], other studies revealed that the induction of Tregs was not changed and even suppressed after infection [7, 27]. These conflicting results could be due to the strain-specific response of the pigs to the virus after challenge or to the method used to evaluate the changes in the Treg response. Several studies have revealed that Tregs are induced due to PRRSV infection, but most of these studies were performed using in vitro or ex vivo assays in which PBMC and mononuclear cells isolated from the lungs and lymph nodes of pigs were infected or re-infected with PRRSV and subsequently observed to monitor the proliferation of various T-cell subsets [36, 37, 58]. However, some ex vivo assays have also demonstrated the inability of certain strains of PRRSV to induce Tregs [59]. In the current study, the Treg frequencies were directly evaluated in the peripheral blood, BAL, lung and lymphoid tissues collected from PRRSV-JA142-infected pigs. During the acute phase of infection, the Treg frequencies largely remained unchanged, but suppression was observed in the BAL throughout the infection period and in BLN at 10 dpc. Comparable results were obtained in a previous in vivo study, revealing that the Treg numbers remained unchanged in the PBMC, lymph nodes and tonsils of the infected pigs up to 28 dpc [7]. Similar results were observed in another study, which showed no significant upregulation of Tregs in the lymph nodes and PBMC at the acute phase of PRRSV infection [27]. Furthermore, the decrease in Treg frequencies in JA142-infected pigs could be attributed to phenotypic plasticity, which resulted in most of the naïve CD4^+^ T cells differentiating into effector cells, such as Th1 and Th17, after being antigenically stimulated during the acute infection [60]. A previous study revealed that the influenza virus-infected pigs showed a similar suppression of Tregs in the BAL and lymph nodes and an increased frequency of T-helper cells during the acute phase of infection [23]. Moreover, in some acute infections of mice with lymphocytic choriomeningitis virus (LCMV) and influenza virus, little or no effect on the immune response was observed after the removal of Tregs from mice [61, 62]. Based on the findings from these studies, it can be deduced that Tregs play only a minor role during many acute infections, but further studies are needed to understand the ultimate cause of this decline in the Treg populations, specifically in BAL cells during PRRSV infection.

Cytokine secretion by immune cells plays a major role in protection against invading pathogens or in the induction of pathology [63]. The levels of proinflammatory cytokines have often been associated with the severity of the disease. However, lower mRNA and protein expression levels of proinflammatory cytokines have been associated with protection against viruses in pigs infected with PRRSV strains that typically induce mild to moderate clinical signs [64]. PRRSV has been shown to reduce or suppress IFN-α production during infection [65]. Similarly, in the current study, IFN-α was peripherally suppressed in the infected pigs with increasing viral replication; however, in the BALF, the levels of IFN-α were maintained, even at peak viremia, possibly playing a role in local viral clearance. Similar to our observations, the induction of IFN-α with increasing viremia has also been observed locally in lymphoid tissues and the BALF in PRRSV-infected pigs [66]. Moreover, local induction of the proinflammatory cytokines IL-1β and IL-8 in lymphoid tissues is reportedly linked to the clearance of PRRSV from infected pigs [67]. Similar induction of the proinflammatory cytokines Il-1α, IL-6, IL-12, and TNF-α has been observed in local tissues in previous studies [66]. In the current study, the induction of proinflammatory cytokines locally in the BALF at peak viremia could thus be linked to their contribution to the clearance of the virus from the pigs. In contrast, the induction of IL-10 in local tissues during early infection was observed in the present study. The production of IL-10, which has been previously observed, reportedly contributes to the persistence of PRRSV by suppressing the immune response in pigs [68]. However, IL-10 production also protects pigs from the tissue damage caused by proinflammatory cytokine overexpression [14]. Tregs are thought to produce IL-10, but in the current study, the Treg frequencies did not change significantly. However, in addition to Tregs, monocytes, Th2 cells, mast cells and B cells are also known sources of IL-10 [69]. Consequently, measuring the systemic cytokine response might not provide a full picture of the protective events orchestrated by cytokines locally in the lungs of PRRSV-infected pigs.

**Figure 11:**
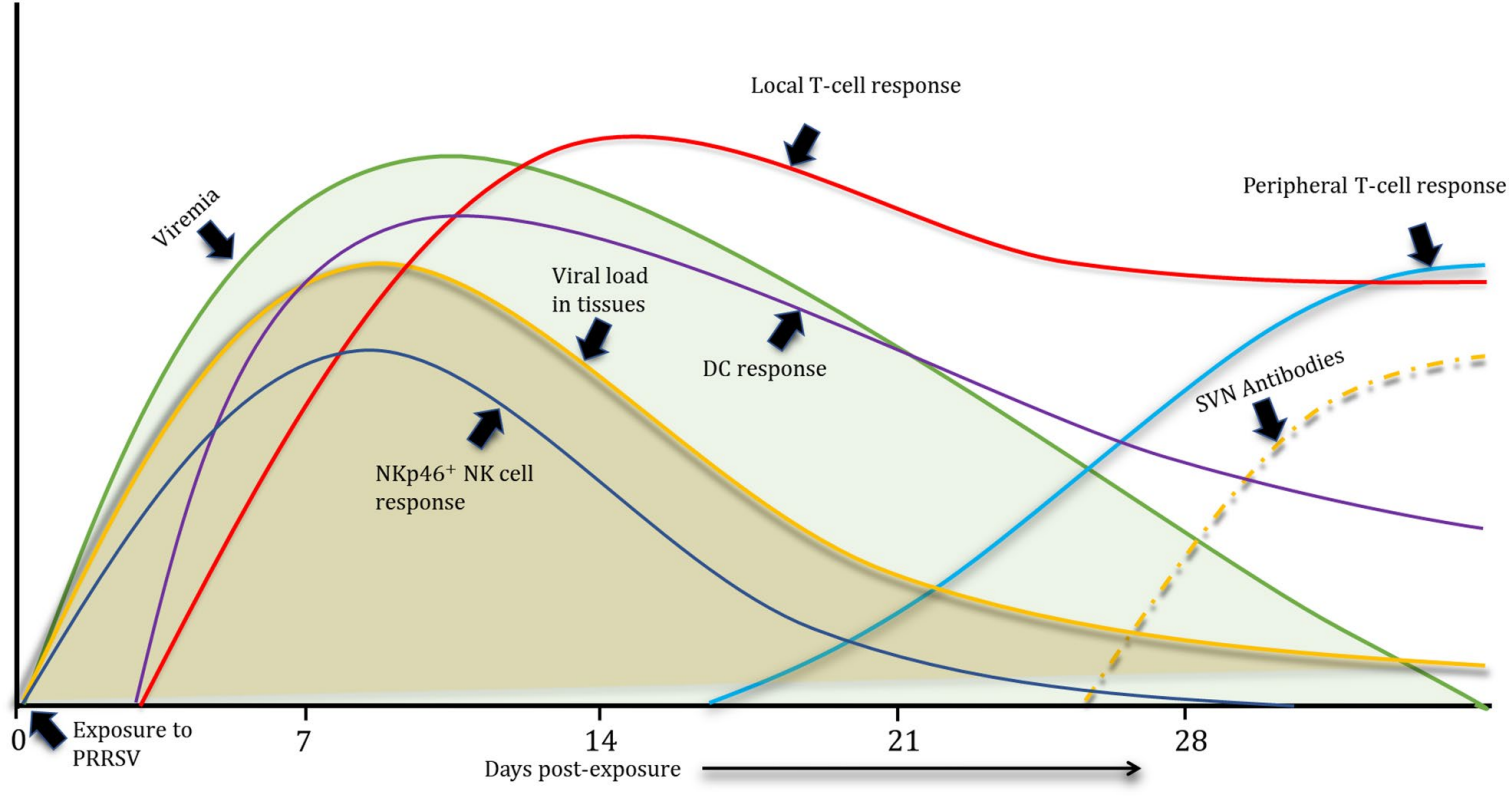
**Chronological order of the immune responses elicited in PRRSV-infected pigs.** This figure shows an overall representation of the immune responses elicited in PRRSV-infected pigs during the acute phase of infection, which is the stage when the virus reached its maximum titre. Most of the virus is cleared from blood and tissues by 28 dpc due to the pig immune responses. The delayed induction of serum virus-neutralizing antibodies and the T-cell response in peripheral blood at 21 days post-exposure, when most of the virus was cleared from the body, indicate that, in addition to the other factors, the early induced local T-cell response plays a key role in the clearance of the virus.

In conclusion, the early stimulation of the DC/macrophage frequencies coincided with the induction of a protective T-cell response in local lymphoid tissues and lung parenchyma, which are known sites of PRRSV replication. Moreover, the local proinflammatory cytokine responses at peak viremia augmented the clearance of the virus from the infected pigs. A temporal sequence of immunobiological events after PRRSV infection in pigs is proposed in Figure 11, and the observed schematic suggests that local T lymphocytes might play an important role in the clearance of the virus from pigs during the acute phase of infection. In addition, early NK cell induction and delayed T-cell responses in peripheral blood were also perceived, and these might play a complementary role in viral clearance. Future studies are needed to further elucidate and refine the understanding of the anti-PRRSV immune response in pigs, and these future studies should primarily focus on obtaining a better understanding of and deciphering the local immune responses with the aim of developing an effective strategy to restrain the virus.

## Supplementary information

**Additional file 1. List of antibodies used in the flow cytometry analysis.**

**Additional file 2. Figures indicating the gating strategies used for various cell subsets in the flow cytometric analysis.**

**Additional file 3. Tabular representation of the percentages of various cell subsets in the PBMC, lung, BAL and BLN samples from infected and uninfected pigs.**

## Data Availability

All the data generated or analysed during the study are included in this published article. The datasets used and/or analysed during the present research project are available from the corresponding author upon reasonable request.

## Abbreviations

ADWG: average daily weight gain
AMs: alveolar macrophages
BAL: bronchoalveolar lavage
BALF: bronchoalveolar lavage fluid
BLNs: bronchial lymph nodes
cDC1s: conventional dendritic cells 1
cDC2s: conventional dendritic cells 2
CPE: cytopathic effect
DMEM: Dulbecco’s modified Eagle’s medium
dpc: days post-challenge
FACS: fluorescence-activated cell sorting
FFN: fluorescent focus neutralization
FoxP3: forkhead box P3
FFU: fluorescent focus unit
IFN: interferon
IL: interleukin
MHC: major histocompatibility complex
moDCs: monocyte-derived dendritic cells
moMɸs: monocyte-derived macrophages
PRRSV: porcine reproductive and respiratory syndrome virus
PRRS: porcine reproductive and respiratory syndrome
PBMCs: peripheral blood mononuclear cells
PBS: phosphate-buffered saline
PI: propidium iodide
RT-qPCR: quantitative real-time reverse transcription PCR
ELISA: enzyme-linked immunosorbent assay
RPMI: Roswell Park Memorial Institute
SVN: serum virus-neutralizing
TNF-α: tumour necrosis factor-alpha
mAbs: monoclonal antibodies
RBCs: red blood cells
TCID: tissue culture infective dose
Th1: T helper 1
Th17: T helper 17
CTLs: cytotoxic T lymphocytes
Tregs: regulatory T cells
