# Supplementary material for: Evaluation of local and systemic immune responses in pigs experimentally challenged with porcine reproductive and respiratory syndrome virus
Source: Vet Res. 2020 May 13;51:66. doi: 10.1186/s13567-020-00789-7 (PMC7222343; doi:10.1186/s13567-020-00789-7)
Supplement: Supplementary file 1 — Additional file 1. List of antibodies used in the flow cytometry analysis. [file 13567_2020_789_MOESM1_ESM.docx]

**Additional file 1 List of antibodies used in the flow cytometry analysis**

| **Name of the primary**  **antibody** | **Mab*/**  **Conjugated Ab** | **Conjugated**  **secondary Ab (α-Mab)** | **Isotype** | **Clone** | **Reference^a^** |
| --- | --- | --- | --- | --- | --- |
| CD4α | PerCP-Cy^TM^5.5 | - | IgG2b | 74-12-4 | 561474 |
| CD8α | PE | - | IgG2a | 76-2-11 | 559584 |
| CD25 | MAb | APC (IgG1) | IgG1 | K231.3B2 | MCA1736GA |
| FoxP3 | FITC | - | IgG2a | FJK-16s | 11-5773-82 |
| CD4α | PE | - | IgG2b | 74-12-4 | 559586 |
| CD8α | FITC | - | IgG2a | 76-2-11 | 551303 |
| IFN- γ | PercpCy5.5 | - | IgG1 | P2G10 | 561481 |
| IL-17 | APC | - | IgG1 | eBio64DEC17 | 17-7179 |
| CD3 EPSILON | FITC | - | IgG1 | PPT3 | MCA5951F |
| CD335/ NKp46 | MAb | APC (IgG1) | IgG1 | VIV-KM1 | MCA5972GA |
| CD163 | MAb | FITC (IgG1) | IgG1 | 2A10/11 | MCA2311GA |
| MHC-II | MAb | PerCP/Cy5.5 (IgG2a) | IgG2a | MSA3 | WS0589S-100 |
| CD172a/SWC3 | MAb | PE (IgG2b) | Mouse (BALB/c)IgG2b,κ | 74-22-15A | 561499 |
| **Name of the secondary antibody** | | | | **Clone** | **Reference^a^** |
| APC anti-mouse IgG1 antibody | | | | RMG1-1 | 406610 |
| FITC anti-mouse IgG1 | | | | RMG1-1 | 406606 |
| PerCP/Cy5.5 anti-mouse IgG2a | | | | RMG2a-62 | 407112 |
| PE anti-mouse IgG2b | | | | RMG2b-1 | 406708 |
| **Name of isotype antibody** | | | | **Clone** | **Reference^a^** |
| Purified mouse IgG2a,κ isotype ctrl antibody | | | | MOPC-173 | 400202 |
| Purified mouse IgG1,κ isotype ctrl antibody | | | | MG1-45 | 401402 |
| Purified mouse IgG2b,κ isotype ctrl antibody | | | | MPC-11 | 400302 |

**Note:** *Mab – monoclonal antibody ^a^ Reference- company catalogue number.
