# Supplementary material for: Evaluation of local and systemic immune responses in pigs experimentally challenged with porcine reproductive and respiratory syndrome virus
Source: Vet Res. 2020 May 13;51:66. doi: 10.1186/s13567-020-00789-7 (PMC7222343; doi:10.1186/s13567-020-00789-7)
Supplement: Supplementary file 2 — Additional file 2. Figures indicating the gating strategies used for various cell subsets in the flow cytometric analysis. [file 13567_2020_789_MOESM2_ESM.pptx]

## Slide 1
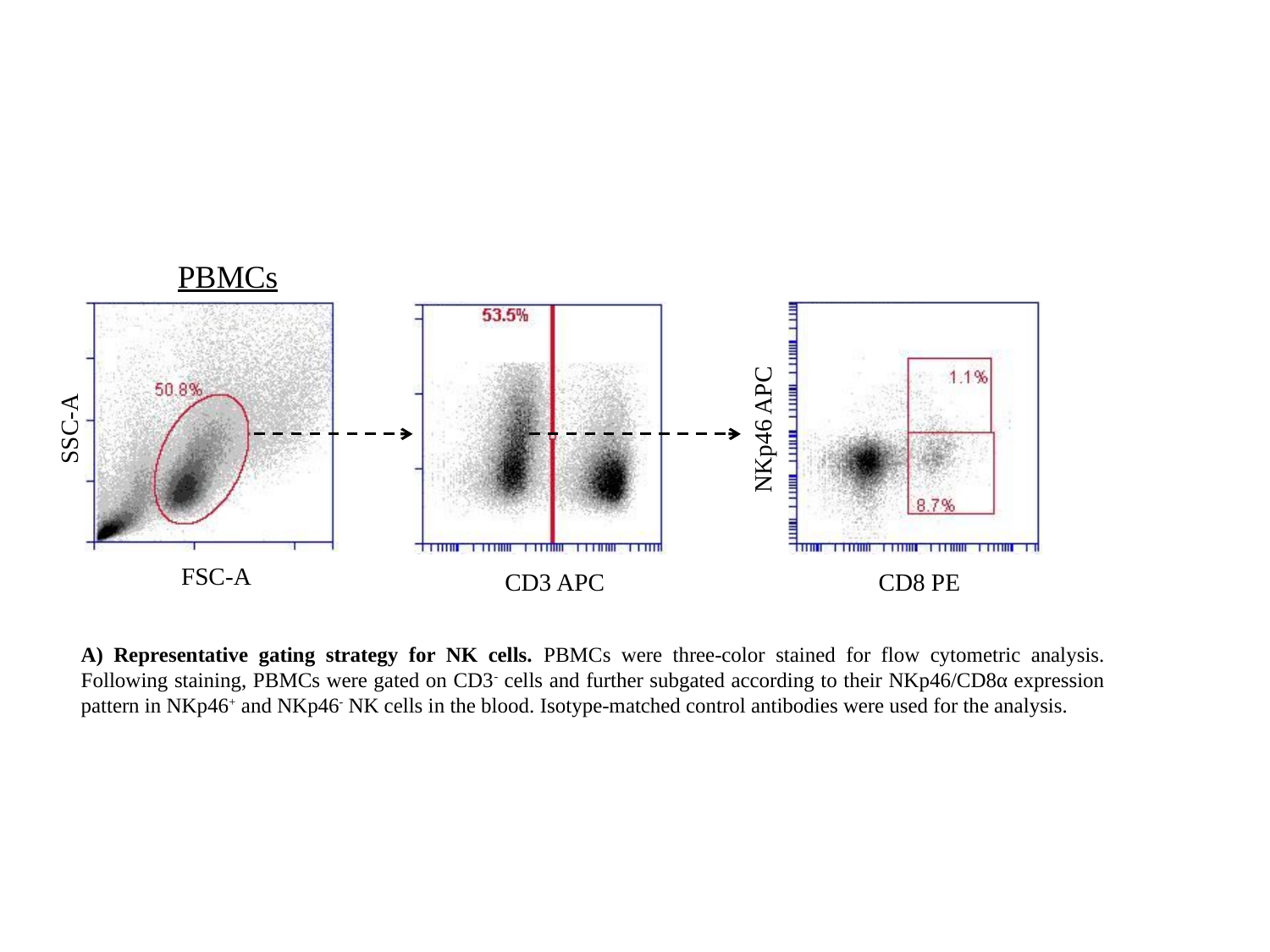

PBMCs
SSC-A
NKp46 APC
FSC-A
CD8 PE
CD3 APC
A) Representative gating strategy for NK cells. PBMCs were three-color stained for flow cytometric analysis. Following staining, PBMCs were gated on CD3- cells and further subgated according to their NKp46/CD8α expression pattern in NKp46+ and NKp46- NK cells in the blood. Isotype-matched control antibodies were used for the analysis.

## Slide 2
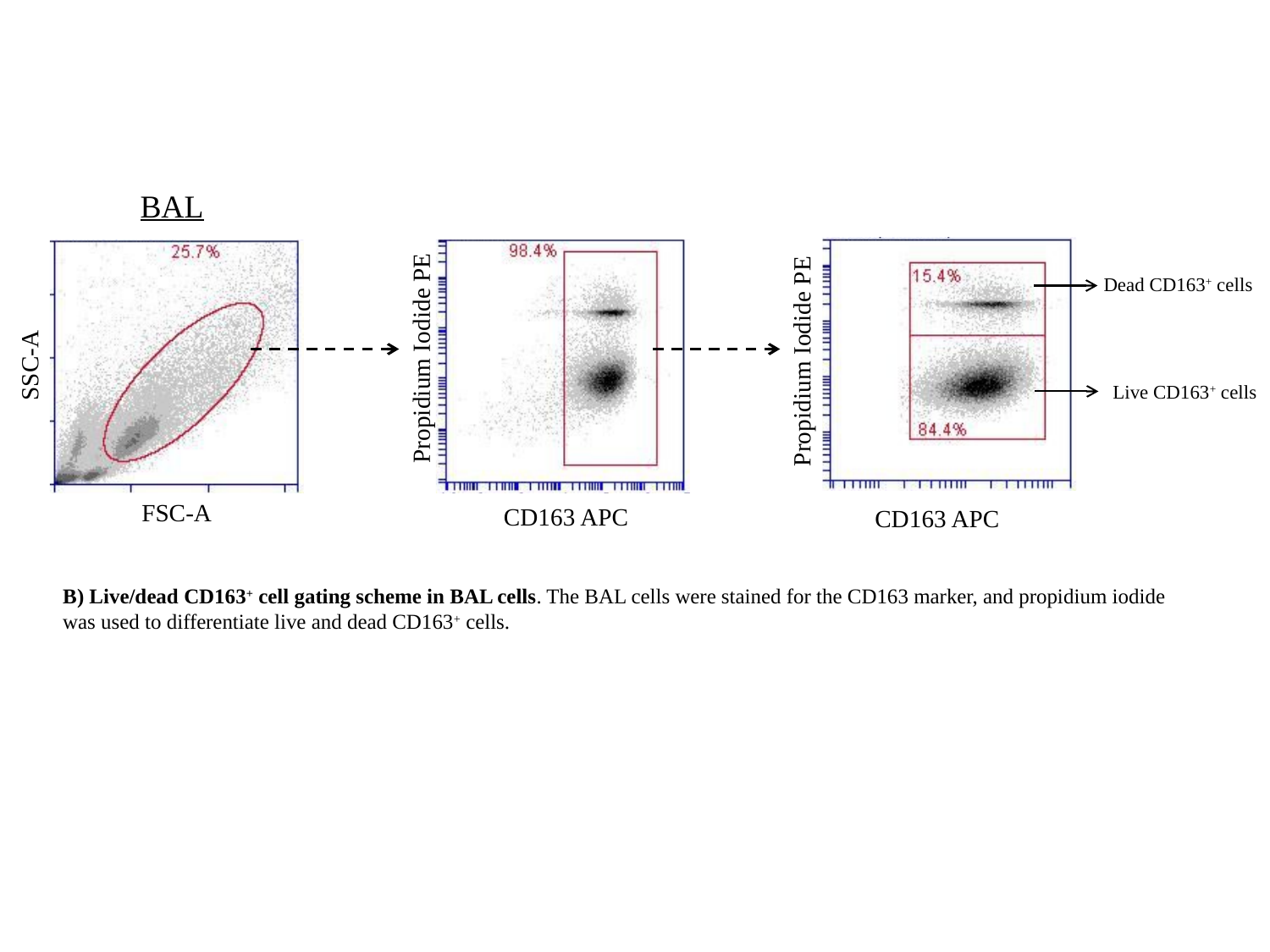

BAL
Dead CD163+ cells
Propidium Iodide PE
Propidium Iodide PE
SSC-A
Live CD163+ cells
FSC-A
CD163 APC
CD163 APC
B) Live/dead CD163+ cell gating scheme in BAL cells. The BAL cells were stained for the CD163 marker, and propidium iodide was used to differentiate live and dead CD163+ cells.

## Slide 3
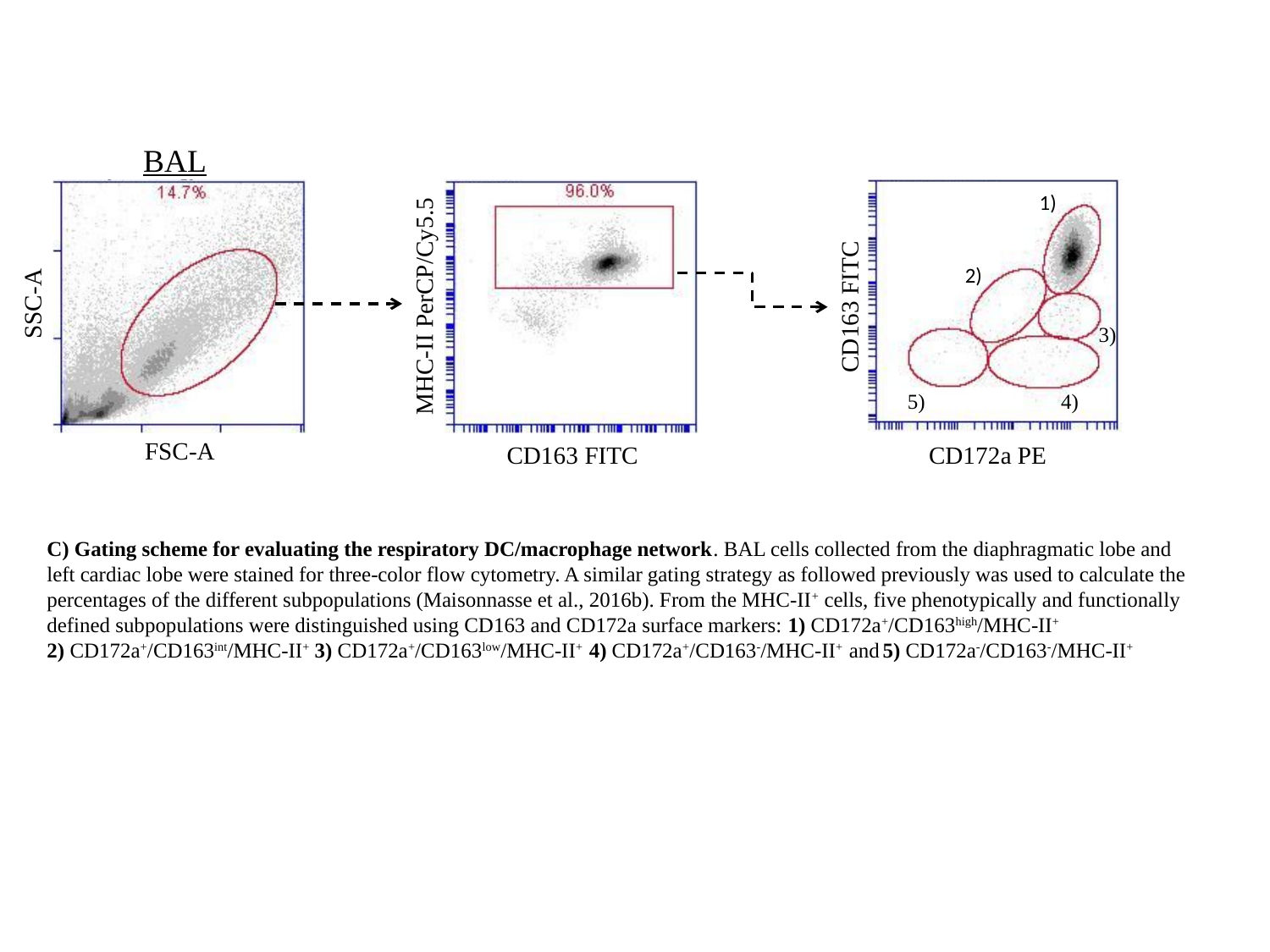

BAL
1)
2)
SSC-A
MHC-II PerCP/Cy5.5
CD163 FITC
3)
5)
4)
FSC-A
CD163 FITC
CD172a PE
C) Gating scheme for evaluating the respiratory DC/macrophage network. BAL cells collected from the diaphragmatic lobe and left cardiac lobe were stained for three-color flow cytometry. A similar gating strategy as followed previously was used to calculate the percentages of the different subpopulations (Maisonnasse et al., 2016b). From the MHC-II+ cells, five phenotypically and functionally defined subpopulations were distinguished using CD163 and CD172a surface markers: 1) CD172a+/CD163high/MHC-II+
2) CD172a+/CD163int/MHC-II+ 3) CD172a+/CD163low/MHC-II+ 4) CD172a+/CD163-/MHC-II+ and 5) CD172a-/CD163-/MHC-II+

## Slide 4
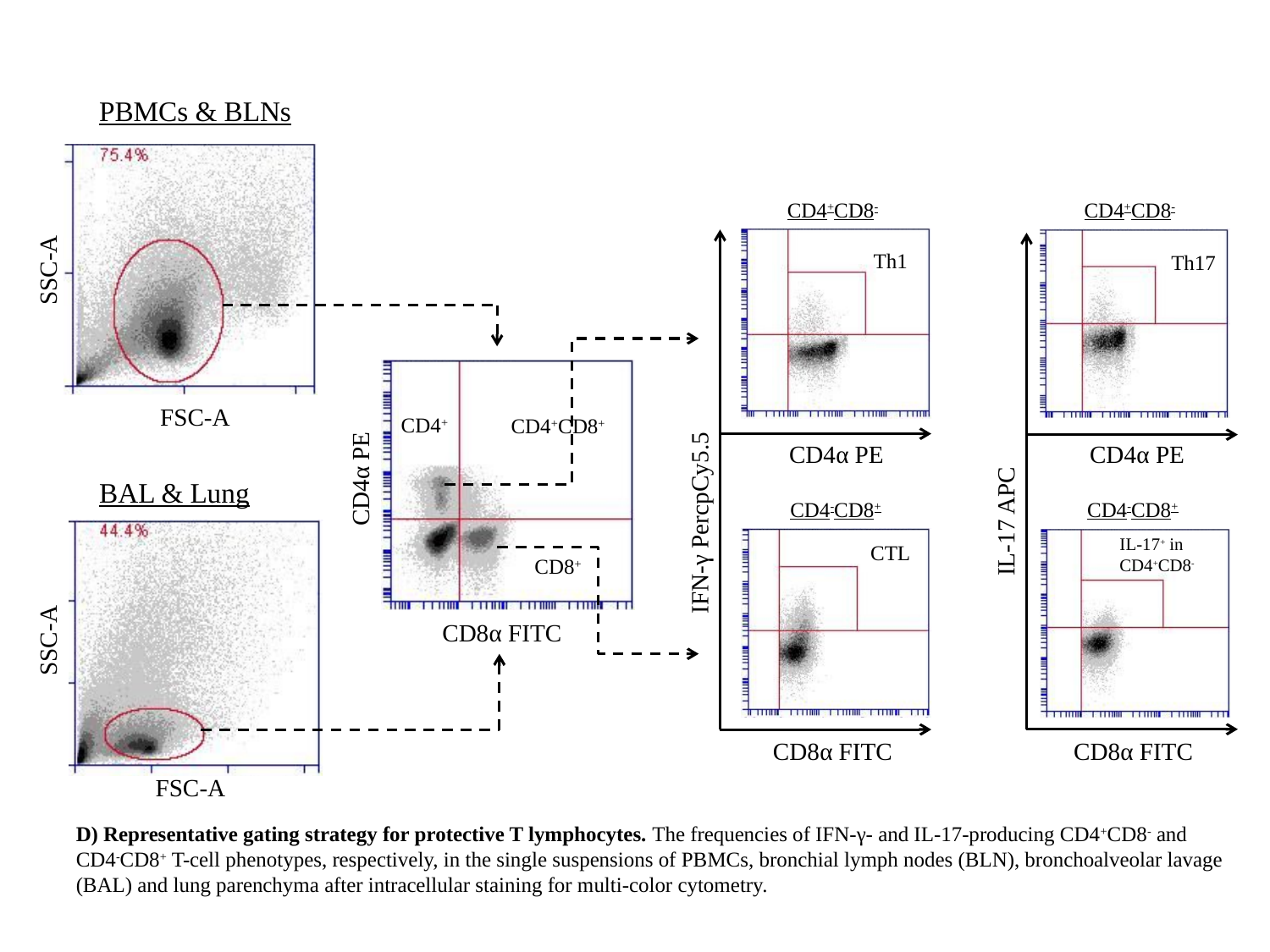

PBMCs & BLNs
CD4+CD8-
CD4+CD8-
Th1
Th17
SSC-A
FSC-A
CD4+
CD4+CD8+
CD4α PE
CD4α PE
CD4α PE
BAL & Lung
CD4-CD8+
CD4-CD8+
IL-17 APC
IFN-γ PercpCy5.5
IL-17+ in CD4+CD8-
CTL
CD8+
CD8α FITC
SSC-A
CD8α FITC
CD8α FITC
FSC-A
D) Representative gating strategy for protective T lymphocytes. The frequencies of IFN-γ- and IL-17-producing CD4+CD8- and CD4-CD8+ T-cell phenotypes, respectively, in the single suspensions of PBMCs, bronchial lymph nodes (BLN), bronchoalveolar lavage (BAL) and lung parenchyma after intracellular staining for multi-color cytometry.

## Slide 5
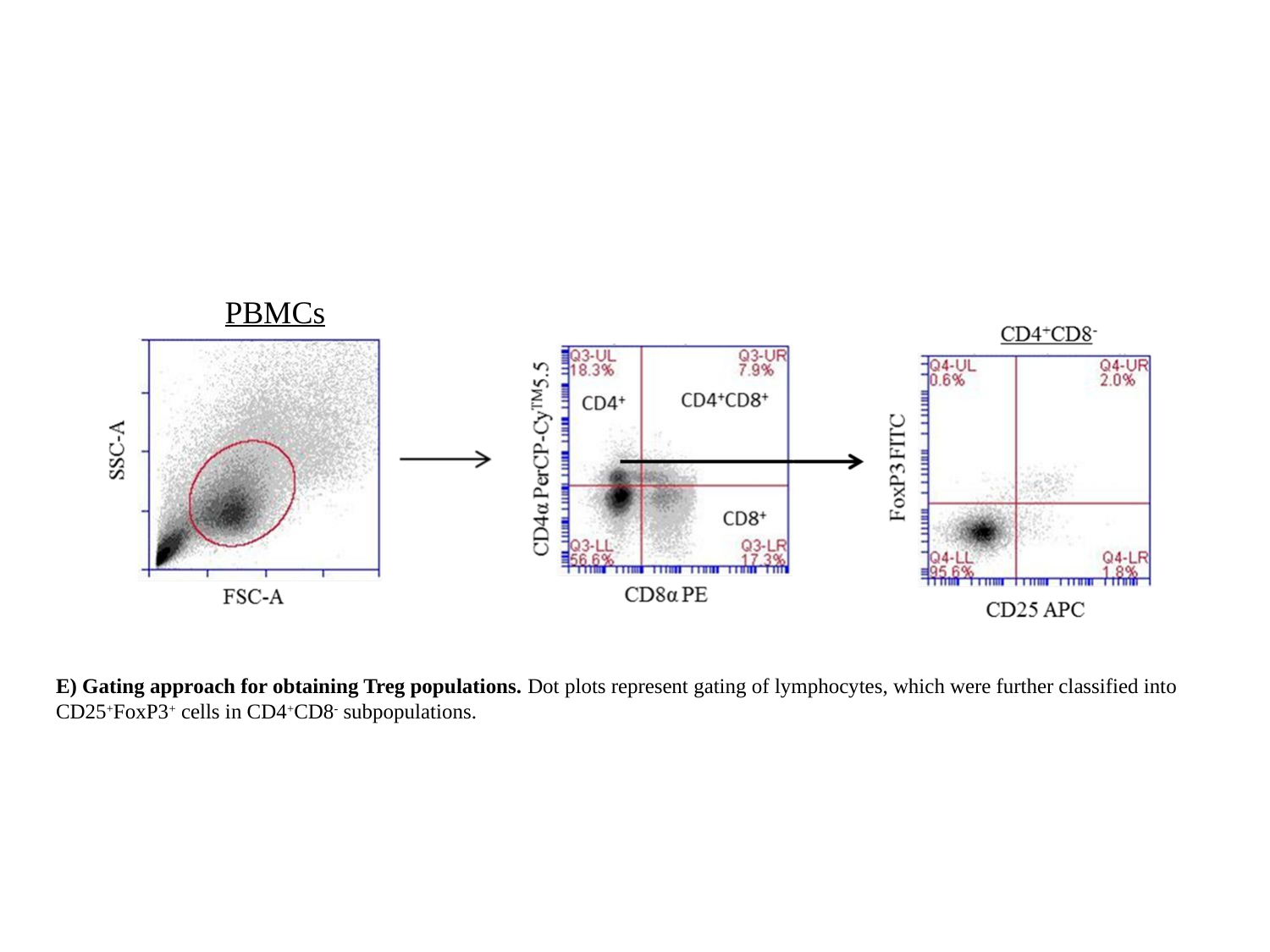

PBMCs
E) Gating approach for obtaining Treg populations. Dot plots represent gating of lymphocytes, which were further classified into CD25+FoxP3+ cells in CD4+CD8- subpopulations.
