# Supplementary material for: Evaluation of local and systemic immune responses in pigs experimentally challenged with porcine reproductive and respiratory syndrome virus
Source: Vet Res. 2020 May 13;51:66. doi: 10.1186/s13567-020-00789-7 (PMC7222343; doi:10.1186/s13567-020-00789-7)
Supplement: Supplementary file 3 — Additional file 3. Tabular representation of the percentages of various cell subsets in the PBMC, lung, BAL and BLN samples from infected and uninfected pigs. [file 13567_2020_789_MOESM3_ESM.docx]

|  | **NC**  (7dpc–35dpc) | | **7 dpc** | | **14 dpc** | | **21 dpc** | | | **28 dpc** | | **35 dpc** | |
| --- | --- | --- | --- | --- | --- | --- | --- | --- | --- | --- | --- | --- | --- |
|  | **Mean** | **SD** | **Mean** | **SD** | **Mean** | **SD** | **Mean** | **SD** | | **Mean** | **SD** | **Mean** | **SD** |
| **CD3^-^ cells** | 47.97 | 9.131 | 46.01 | 6.741 | 54.42 | 7.900 | 48.57 | | 4.988 | 50.06 | 6.150 | 45.23 | 5.625 |
| **NKp46^+^ NK cells**  (NKp46^+^CD8^+^ in CD3^-^) | 0.436 | 0.4552 | 2.753 | 1.974 | 2.027 | 2.542 | 0.296 | | 0.1289 | 0.266 | 0.1540 | 0.335 | 0.1886 |
| **NKp46^-^ NK cells**  (NKp46^-^CD8^+^ in CD3^-^) | 13.65 | 6.327 | 15.56 | 9.810 | 15.46 | 7.426 | 15.94 | | 6.320 | 18.86 | 6.442 | 20.06 | 7.191 |
| **CD4^+^CD8^-^**  **T cells** | 13.81 | 3.397 | 13.29 | 4.859 | 11.85 | 3.853 | 15.71 | | 2.348 | 11.67 | 2.595 | 11.84 | 2.295 |
| **CD4^-^CD8^+^**  **T cells** | 20.01 | 9.205 | 32.49 | 7.609 | 31.36 | 5.885 | 25.19 | | 6.070 | 30.86 | 8.886 | 35.59 | 8.379 |
| **Th1 cells**  (IFN-γ^+^ in CD4^+^CD8^-^) | 4.777 | 1.795 | 4.682 | 2.126 | 6.308 | 2.887 | 7.747 | | 3.131 | 7.124 | 3.206 | 7.206 | 4.657 |
| **CTL**  (IFN-γ^+^ in CD4^-^CD8^+^) | 16.39 | 4.900 | 16.82 | 5.169 | 18.97 | 5.400 | 19.21 | | 4.947 | 16.96 | 4.758 | 19.34 | 4.946 |
| **Th17 cells**  (IL17^+^ in CD4^+^CD8^-^) | 3.630 | 1.342 | 3.481 | 1.601 | 3.777 | 2.317 | 5.506 | | 2.024 | 6.065 | 2.549 | 10.92 | 4.006 |
| **IL17^+^ in**  **CD4^-^CD8^+^ cells** | 5.134 | 2.073 | 4.713 | 1.935 | 4.979 | 3.098 | 6.601 | | 1.980 | 6.989 | 2.291 | 6.264 | 1.682 |
| **CD4^+^CD8^-^**  **Tregs** | 4.053 | 1.295 | 3.492 | 1.080 | 4.151 | 1.648 | 3.957 | | 0.8119 | 4.473 | 0.9257 | 2.471 | 0.8383 |

**Additional file 3A Percentage of different immune cells in PBMCs of PRRSV infected and uninfected pigs**

|  | **NC**  (10dpc–35dpc) | | **10 dpc** | | **21 dpc** | | **28 dpc** | | **35 dpc** | |
| --- | --- | --- | --- | --- | --- | --- | --- | --- | --- | --- |
|  | **Mean** | **SD** | **Mean** | **SD** | **Mean** | **SD** | **Mean** | **SD** | **Mean** | **SD** |
| **CD4^+^CD8^-^**  **T cells** | 4.574 | 1.777 | 6.070 | 1.581 | 5.506 | 1.395 | 5.174 | 1.985 | 4.253 | 0.7273 |
| **CD4^-^CD8^+^**  **T cells** | 44.91 | 11.93 | 53.86 | 8.128 | 48.23 | 4.744 | 42.60 | 7.945 | 59.33 | 6.016 |
| **Th1 cells**  (IFN-γ^+^ in CD4^+^CD8^-^) | 5.260 | 2.465 | 23.90 | 5.838 | 14.12 | 7.764 | 7.879 | 5.292 | 8.247 | 5.090 |
| **CTL**  (IFN-γ^+^ in CD4^-^CD8^+^) | 11.37 | 4.640 | 17.72 | 5.166 | 16.06 | 6.387 | 10.33 | 3.648 | 12.25 | 5.433 |
| **Th17 cells**  (IL17^+^ in CD4^+^CD8^-^) | 2.921 | 1.684 | 15.43 | 3.931 | 9.449 | 5.870 | 3.935 | 2.985 | 4.769 | 3.378 |
| **IL17^+^ in**  **CD4^-^CD8^+^ cells** | 1.181 | 0.6752 | 5.243 | 2.007 | 3.018 | 1.614 | 1.799 | 0.8358 | 1.475 | 1.165 |
| **CD4^+^CD8^-^**  **Tregs** | 1.655 | 0.7583 | 0.8479 | 0.2989 | 1.419 | 0.7130 | 1.704 | 1.118 | 1.973 | 0.6369 |

**Additional file 3B Percentage of T-cell subsets in negative and PRRSV infected pigs in lungs**

|  | **NC** | | **3** **dpc** | | **10** **dpc** | | **21 dpc** | | **28 dpc** | | **35 dpc** | |
| --- | --- | --- | --- | --- | --- | --- | --- | --- | --- | --- | --- | --- |
|  | **Mean** | **SD** | **Mean** | **SD** | **Mean** | **SD** | **Mean** | **SD** | **Mean** | **SD** | **Mean** | **SD** |
| **CD4^+^CD8^-^ T cells** | 10.26 | 4.878 | **nt*** | | 10.61 | 5.275 | 5.503 | 1.234 | 5.192 | 1.127 | 4.694 | 1.298 |
| **CD4^-^CD8^+^ T cells** | 37.66 | 8.255 | **nt** | | 37.67 | 10.13 | 68.09 | 7.379 | 50.68 | 10.22 | 52.14 | 9.465 |
| **Th1 cells**  (IFN-γ^+^ in CD4^+^CD8^-^) | 5.922 | 2.820 | **nt** | | 12.72 | 4.289 | 27.76 | 10.45 | 13.77 | 6.702 | 11.70 | 4.766 |
| **CTL**  (IFN-γ^+^ in CD4^-^CD8^+^) | 8.989 | 4.239 | **nt** | | 12.79 | 3.047 | 22.29 | 10.33 | 14.30 | 4.885 | 11.66 | 3.792 |
| **Th17 cells**  (IL17^+^ in CD4^+^CD8^-^) | 2.261 | 1.042 | **nt** | | 4.071 | 1.361 | 13.49 | 6.858 | 5.522 | 3.395 | 4.728 | 2.575 |
| **IL17^+^ in**  **CD4^-^CD8^+^ cells** | 1.635 | 0.9809 | **nt** | | 2.948 | 1.066 | 4.216 | 3.313 | 1.627 | 0.9029 | 1.619 | 0.8125 |
| **CD4^+^CD8^-^**  **Tregs** | 2.110 | 0.6697 | **nt** | | 0.493 | 0.2520 | 3.728 | 1.508 | 0.749 | 0.3730 | 0.915 | 0.4533 |
| CD172a^+^CD163^high^/  MHC-II ^+^ | 98.13 | 2.202 | 99.38 | 0.4988 | 85.00 | 5.996 | 93.79 | 3.395 | 95.38 | 3.420 | 97.89 | 1.117 |
| CD172a^+^CD163^int^/ MHC-II ^+^ | 0.390 | 0.3792 | 0.254 | 0.3351 | 5.814 | 2.990 | 1.969 | 1.520 | 1.714 | 2.557 | 1.344 | 0.7253 |
| CD172a^+^CD163^low^/  MHC-II ^+^ | 0.789 | 1.040 | 0.133 | 0.0706 | 8.435 | 5.206 | 2.808 | 1.934 | 1.706 | 1.039 | 0.253 | 0.1849 |
| CD172a^-^ CD163/  MHC-II^+^ | 0.018 | 0.0218 | 0.009 | 0.0079 | 0.534 | 0.4697 | 0.244 | 0.4073 | 0.109 | 0.0855 | 0.043 | 0.0506 |
| CD172a^+^ CD163/  MHC-II^+^) | 0.115 | 0.1442 | 0.027 | 0.0148 | 0.987 | 0.7040 | 0.191 | 0.1476 | 0.241 | 0.1662 | 0.017 | 0.0253 |
| ***nt: not tested** | | | | | | | | | | | | |

**Additional file 3C Percentage of immune cells in BAL of uninfected and PRRSV infected pigs**

**Additional file 3D Percentage of T-cell subsets in negative and PRRSV infected pigs in lymph nodes**

|  | **NC**  (10dpc–35dpc) | | **10 dpc** | | **21 dpc** | | **28 dpc** | | **35 dpc** | |
| --- | --- | --- | --- | --- | --- | --- | --- | --- | --- | --- |
|  | **Mean** | **SD** | **Mean** | **SD** | **Mean** | **SD** | **Mean** | **SD** | **Mean** | **SD** |
| **CD4^+^CD8^-^**  **T cells** | 24.18 | 6.261 | 20.31 | 4.572 | 26.19 | 5.016 | 26.39 | 6.451 | 19.00 | 4.548 |
| **CD4^-^CD8^+^**  **T cells** | 18.33 | 3.815 | 12.04 | 2.683 | 16.99 | 4.152 | 22.62 | 3.690 | 22.94 | 2.343 |
| **Th1 cells**  (IFN-γ^+^ in CD4^+^CD8^-^) | 3.093 | 1.195 | 8.620 | 3.122 | 9.093 | 2.796 | 6.704 | 2.120 | 6.764 | 2.091 |
| **CTL**  (IFN-γ^+^ in CD4^-^CD8^+^) | 8.759 | 1.912 | 13.24 | 5.566 | 15.31 | 2.581 | 14.35 | 5.137 | 14.85 | 4.113 |
| **Th17 cells**  (IL17^+^ in CD4^+^CD8^-^) | 1.148 | 0.4577 | 2.811 | 1.372 | 3.076 | 1.082 | 1.756 | 0.5015 | 2.972 | 1.029 |
| **IL17^+^ in**  **CD4^-^CD8^+^ cells** | 2.428 | 0.7090 | 3.671 | 1.812 | 5.125 | 1.212 | 4.256 | 1.635 | 3.212 | 0.9930 |
| **CD4^+^CD8^-^**  **Tregs** | 8.770 | 3.105 | 5.003 | 1.894 | 7.339 | 2.053 | 11.05 | 3.075 | 10.27 | 2.470 |
